# Marine Fungal Diversity and Dynamics in the Gulf of Trieste (Northern Adriatic Sea)

**DOI:** 10.1007/s00248-024-02394-z

**Published:** 2024-05-29

**Authors:** Elisa Banchi, Vincenzo Manna, Lucia Muggia, Mauro Celussi

**Affiliations:** 1 National Institute of Oceanography and Applied Geophysics (OGS), Trieste, Italy; 2 NBFC, National Biodiversity Future Center, Palermo, Italy; 3 Department of Life Sciences, University of Trieste, Trieste, Italy

**Keywords:** Amplicon sequencing, DNA metabarcoding, Mycoplankton, Parengyodontium album

## Abstract

Fungi contribute to different important ecological processes, including decomposition of organic matter and nutrient cycling, but in the marine environment the main factors influencing their diversity and dynamics at the spatial and temporal levels are still largely unclear. In this study, we performed DNA metabarcoding on seawater sampled monthly over a year and a half in the Gulf of Trieste (northern Adriatic Sea), targeting the internal transcribed spacer (ITS) and the 18S rRNA gene regions. The fungal communities were diverse, very dynamic, and belonged predominantly to marine taxa. Samples could be clustered in two groups, mainly based on the high (> 30%) or low relative proportion of the ascomycetes *Parengyodontium album*, which emerged as a key taxon in this area. Dissolved and particulate organic C:N ratio played important roles in shaping the mycoplankton assemblages, suggesting that differently bioavailable organic matter pools may be utilized by different consortia. The proportion of fungal over total reads was 31% for ITS and 0.7% for 18S. ITS had the highest taxonomic resolution but low power to detect early divergent fungal lineages. Our results on composition, distribution, and environmental drivers extended our knowledge of the structure and function of the mycobiome of coastal waters.

**Supplementary Information:**

The online version contains supplementary material available at 10.1007/s00248-024-02394-z.

## Introduction

In the marine environment, fungi have been detected in every explored habitat, from surface to deeper waters, from the coast to the open ocean, and from beaches to deep sediments [1, 2]. However, compared to their terrestrial counterparts, planktonic marine fungi, belonging to the mycoplankton, have been much less studied in terms of occurrence, biodiversity, dynamics, and contribution to ecosystem processes [3, 4]. Like terrestrial species, marine fungi are thought to contribute to organic matter degradation processes and nutrient cycling by acting as saprotrophic (i.e., decomposers) or parasitic organisms at different trophic levels [5]. For example, fungal zoospores efficiently transfer organic matter from large, otherwise inedible, phytoplankton cells to zooplankton, in a process termed mycoloop [6]. While mycoplankton are thought to have significant impacts on ecosystems, these organisms remain poorly understood [7].

More than 10,000 marine fungal species are estimated to live in the ocean, although less than 1900 have been formally described to date [8] (https://www.marinefungi.org/). Recently, after debated opinions [5], the scientific community has agreed on a common definition to classify a marine fungus, i.e., “it is repeatedly recovered from marine habitats because it is able to grow and/or sporulate in marine environments, it forms symbiotic relationships with other organisms, or it adapts and evolves at the genetic level or is metabolically active in the marine environment” [5]. While mycoplankton research has traditionally relied mainly on microscopic and culture-based approaches [5], the use of molecular tools is now overcoming the limitations of cultivation-based methods and revealing a large, previously unknown biodiversity [1, 7, 9]. These approaches, nonetheless, offer their own challenges. For example, passive propagule dispersion should be taken into account when assessing the role of fungi in marine habitats [10] when applying culture-independent, DNA-based approaches to estimate fungal diversity in near-shore environments.

Marine fungi have generally been overlooked in the context of global, ground-breaking programmes such as the Global Ocean Sampling expedition [11] and TARA Oceans [12], which have provided unprecedented resources for the genomics of marine prokaryotes and other eukaryotes [9]. One of the main limiting factors for DNA-based assessment of mycoplankton is the bias from "generic" eukaryotic primers (targeting the 18S rRNA gene [13]), which can lead to a lack of amplified fragments and consequently an underestimation of the proportion and diversity of fungi in biological communities [9, 14]. Therefore, the use of fungal-specific barcodes, such as those targeting the ribosomal nuclear gene internal transcribed spacer (ITS) [15] or the 18S rRNA gene [16], can provide greater insight into marine fungi biodiversity, although requiring *ad hoc* amplification, sequencing, and data analysis. In addition, mycoplankton can have patchy and highly variable spatiotemporal distribution patterns compared to bacterioplankton [9, 15, 17], making data analysis and interpretation more complex. Therefore, additional efforts are needed to explore the diversity and ecological role of fungi in marine habitats.

Here, we provide new insights into fungal communities in coastal environments, where mycoplankton are thought to play a particularly important role in organic matter cycling [4]. In this perspective, Long Term Ecological Research (LTER) stations represent a valuable resource and strategic advantage to assess patterns of mycoplankton diversity [15]. At the LTER C1 station (Gulf of Trieste, northern Adriatic Sea), seawater was sampled monthly over 1.5 years, and DNA metabarcoding targeting the ITS1 region was used to investigate mycoplankton composition, distribution, and key environmental factors influencing their temporal patterns, an effort directed to expand current knowledge of the structure and dynamics of coastal marine mycobiome. As a proof of concept, the fungal community was also investigated through 18S V4 rRNA gene metabarcoding to assess and compare the proportion and identity of the taxa identified with the two target regions.

## Methods

### Sample Collection and Environmental Data

**Fig. 1:**
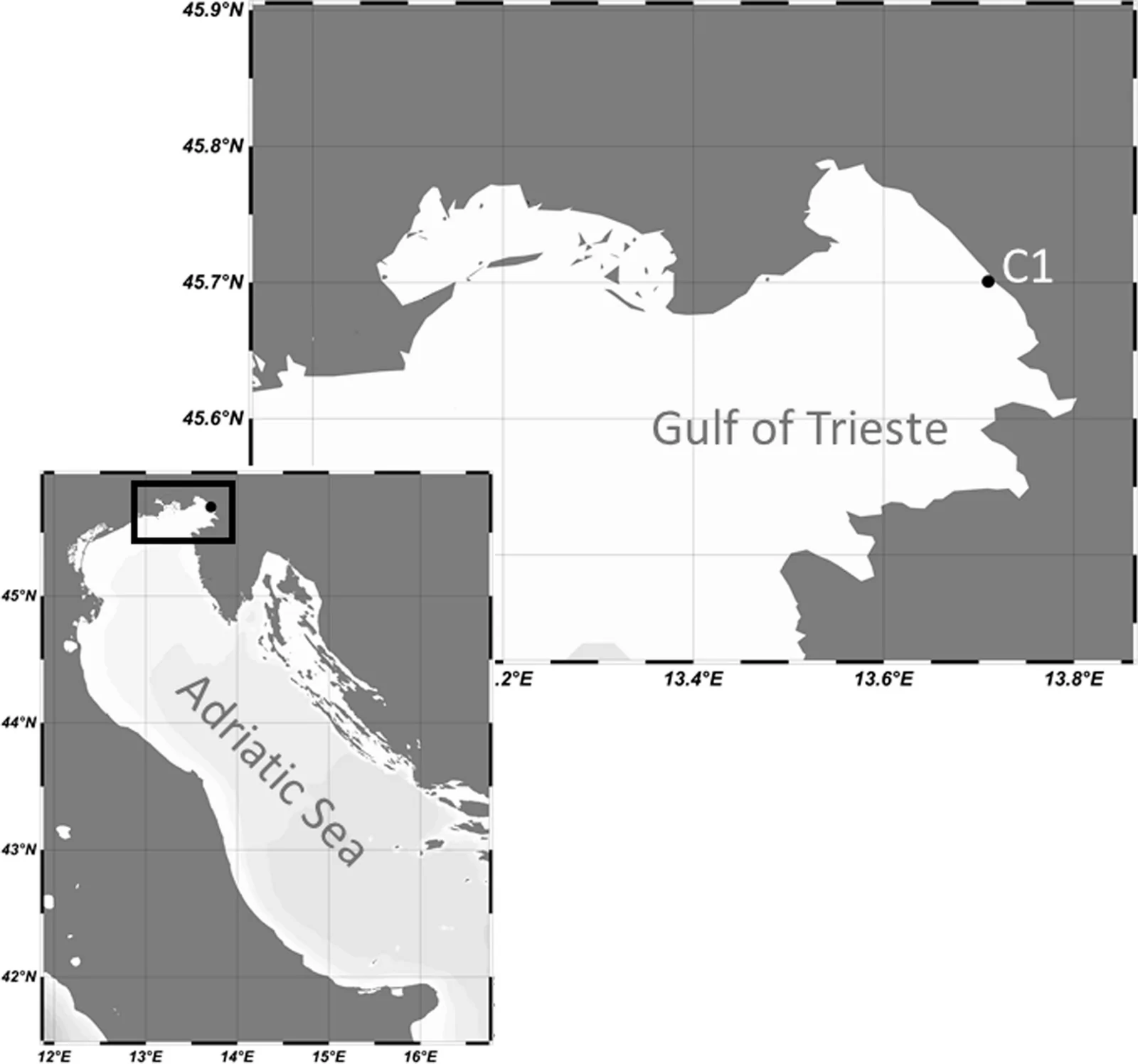
C1 sampling site location in the northern Adriatic Sea. Maps designed with Ocean Data View (https://odv.awi.de)

The sampling site was the LTER monitoring station C1 (45° 42′ 2″ N, 13° 42′ 36″ E; Fig. 1) located in the Gulf of Trieste (northern Adriatic Sea). Seawater samples were collected monthly between October 2018 and April 2020 at the surface (~ 1 m depth, S) and at the bottom (15 m depth, B) of the water column using 5-L Niskin bottles. Samples were filtered through 0.2 μm PES membrane filters (PALL Laboratory) until clogging (1–3 L) and stored at −80 °C until further processing. In the frame of the LTER monitoring at station C1, contextually to DNA sampling, a set of biogeochemical parameters was determined. Temperature and salinity were measured by means of a multiparametric probe (SBE 19plus SEACAT). The concentrations of chlorophyll *a* (Chl*a*), dissolved organic carbon (DOC), particulate organic carbon (POC), dissolved organic nitrogen (DON), and total particulate nitrogen (TPN) were determined according to standard procedures [18, 19] (Supplementary results and Fig. S1).

### DNA Extraction and Amplicon Sequencing

DNA was extracted using the DNeasy PowerWater Kit (Qiagen) with some modifications (two additional vortexing steps for 2 min at the maximum speed, each one preceded by an incubation at 70 °C for 5 min) [20].

For amplicon sequencing, the nuclear ribosomal internal transcribed spacer 1 (ITS1) region was amplified in all samples (Surface and Bottom, for a total of 38 samples) using the primers ITS1-F [21] and ITS2-R [22]. Libraries were prepared following the Illumina Sequencing Library Preparation protocol (with 10 µL of DNA and 35 PCR cycles in the amplicon PCR) and ran on an Illumina MiSeq System for a read length of 2 × 250 bp at Cogentech (Consortium for Genomic Technologies c/o IFOM-IEO Campus, Milano, Italy).

The V4 region of the 18S rRNA (18S V4) gene was amplified only in the surface samples (for a total of 19 samples) using the "generic" eukaryotic primers TAReuk454FWD1 [13] and TAReukREV3_modified [23, 24]. Libraries were prepared following the Illumina Sequencing Library Preparation protocol and ran on an Illumina MiSeq System for a read length of 2 × 250 bp BMR genomics (Padova, Italy).

### Bioinformatic Pipelines

For the ITS1 barcode, the PIPITS v. 3.0 pipeline [25] was used at default parameters for reads merging and quality filtering. ITS1 region was extracted with ITSx [25]. Then, operational taxonomic unit (OTU) clustering at 97% similarity and chimera removal were performed with VSEARCH v. 2.23.0 [25]. OTUs with a frequency < 10 were removed. Alpha-diversity metrics were estimated after samples were rarefied.

Taxonomy was assigned to OTUs within QIIME2 (v. 2023.5) [26] using the sklearn naïve Bayes taxonomy classifier against the UNITE reference database (v. 9) [27]. BLASTN [28] was also used as an additional check, aligning the representative sequences against the nucleotide collection (BLAST + 2.14.1).

Finally, only sequences belonging to fungi (fungi—Kingdom Mycetae—and fungal-like organisms—Oomycetes, Hyphochytriomycetes, Labyrinthulomycetes in Kingdom Straminipila—[29]) were retained in the final dataset. A "core" mycobiome was identified by selecting the genera present in more than 95% of the samples. To assess similarity patterns of fungal communities, a hierarchical clustering of the OTU table (Ward.D2 method) was built using the function *hclust* in the R (v. 4.0.3) [30] package *stats* (v. 4.3.0). A permutational multivariate analysis of variance (PERMANOVA) with 4999 permutations was computed on the normalized OTU table using the function *adonis* in the R (v. 4.0.3) [30] package *vegan* (v. 2.6.4) [31]. To investigate the fungal community environmental drivers, a distance-based redundancy analysis (dbRDA) was performed on Bray-Curtis dissimilarity matrices on the OTU table using the function *capscale* in the R (v. 4.0.3) [30] package *vegan* (v. 2.6.4) [31]. The environmental variables were first screened for collinearity [32], and the model and variable significance were tested with ANOVA and 4999 permutations. Marine genera were assessed following comprehensive works and reviews based on both traditional and molecular methods [2, 8, 10, 33]. Marine genera were assessed considering only taxa that presented > 0.01% of average relative abundance (considered non-rare taxa [34]). FUNGuild [35] was used to predict marine genera trophic and growth modes, functional guilds, and habitat type.

For the 18S V4 barcode, analyses were performed with QIIME2 (v. 2023.5) [26] using DADA2 for denoising. Amplicon sequence variants (ASVs) with a frequency < 2 were removed. Taxonomy was assigned to ASVs using the sklearn naïve Bayes taxonomy classifier against the SILVA 99% reference database (v. 138.1) [36], and only ASVs belonging to Fungi were retained.

## Results

### Fungal Community

The ITS1 amplicon sequencing of the 38 samples generated 5,836,517 raw reads. After cleaning, ITS1 extraction, and chimera removal, 4,528,841 reads were retained with an average of 119,180 ± 59,972 per sample and a total of 6885 OTUs with an average of 1170 ± 577 per sample. Sample Dec19_B was removed as 92.2% of its reads belonged to a cnidaria. After removing the non-fungal sequences (all of them belonging to the Myceteae), 1,396,478 reads (31% of the total dataset) were retained. The total number of OTUs was 2017 with an average of 246 (± 196) per sample. Shannon’s diversity index was, on average, 3.5 (± 1.7), while Pielou’s evenness was 0.50 (± 0.17).

**Fig. 2:**
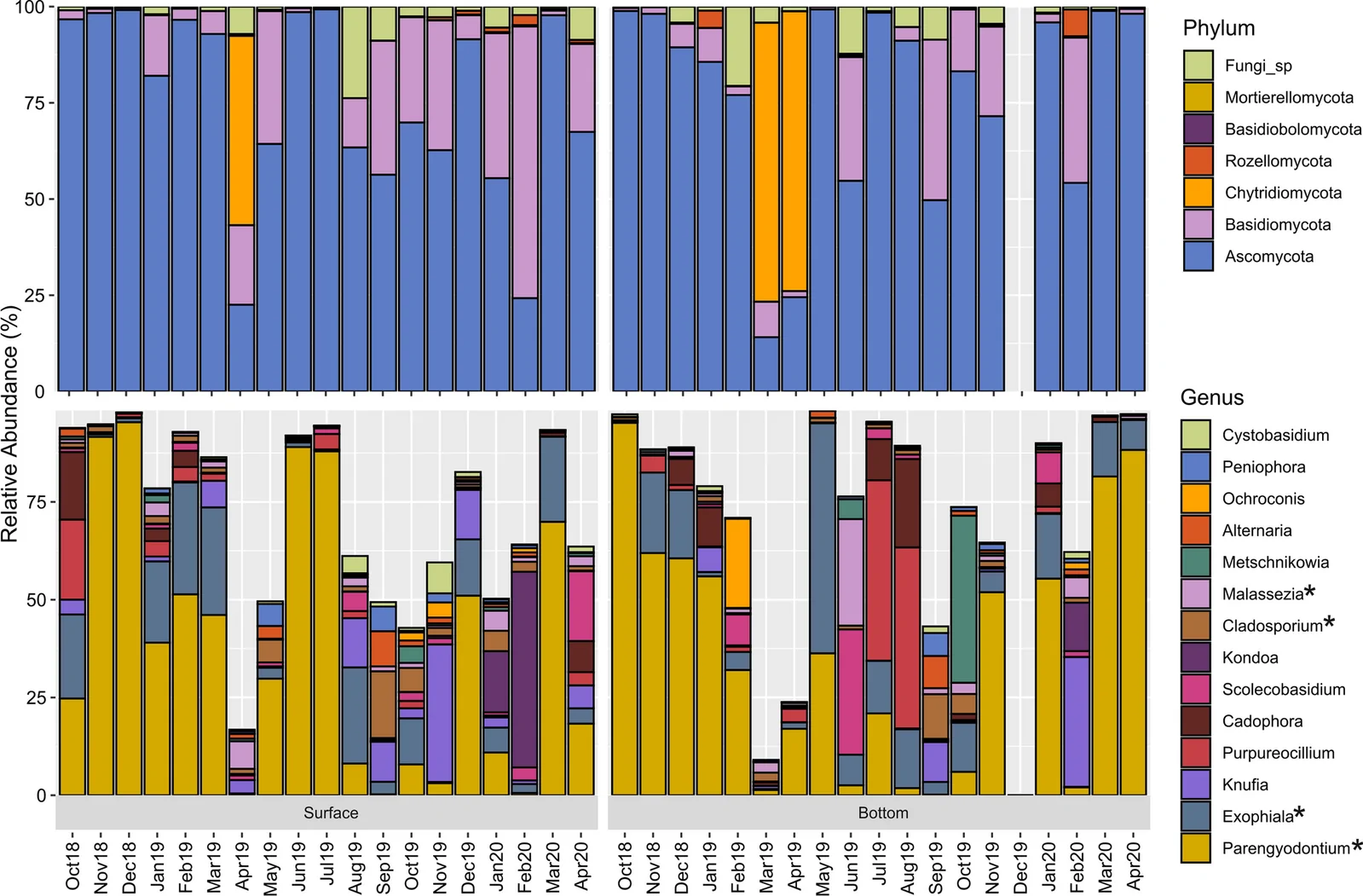
Taxonomic composition of fungi at the phylum (upper panel) and genus (lower panel; average relative abundance > 0.5) levels. Asterisks indicate core genera

The taxonomy of the fungal OTUs in each sample, presented as Krona charts [37], is available at https://github.com/ElisaBanchi/Fungi_ITS. Six phyla were detected, with most reads assigned to Ascomycota (76.5 ± 25.3% on average) (Fig. 2). Ascomycota was generally the dominant phylum (in 34 out of 38 samples; Fig. 2), while Basidiomycota prevailed in a surface winter water sample (Feb20_S; Fig. 2) and Chytridiomycota in early spring (Fig. 2).

A total of 772 genera (of which 653 were identified with a generic name; Table S1) were detected, the most abundant being *Parengyodontium* (a monospecific genus with the only known species *P. album*, 37.2 ± 33.0%; Fig. 2). Only 14 genera reached an average abundance > 0.5% (Fig. 2), while 505 genera could be considered rare (< 0.01%; Table S1).

We identified six genera as representatives of the core mycobiome in the entire data set, i.e., *Aspergillus*, *Cladosporium*, *Exophiala*, *Malassezia*, *Parengyodontium*, and *Penicillium*.

Due to the highly dynamic taxonomic composition across the dataset (Fig. 2), we performed hierarchical clustering to highlight the similarity among samples. Samples fell into two well-distinct clusters (Fig. S2), with Cluster 1 comprising all samples in which the relative proportion of *P. album* was greater than 30% (Fig. S2). PERMANOVA showed that the separation of the clusters explained 82% of the variance (*p* < 0.001).

**Fig. 3:**
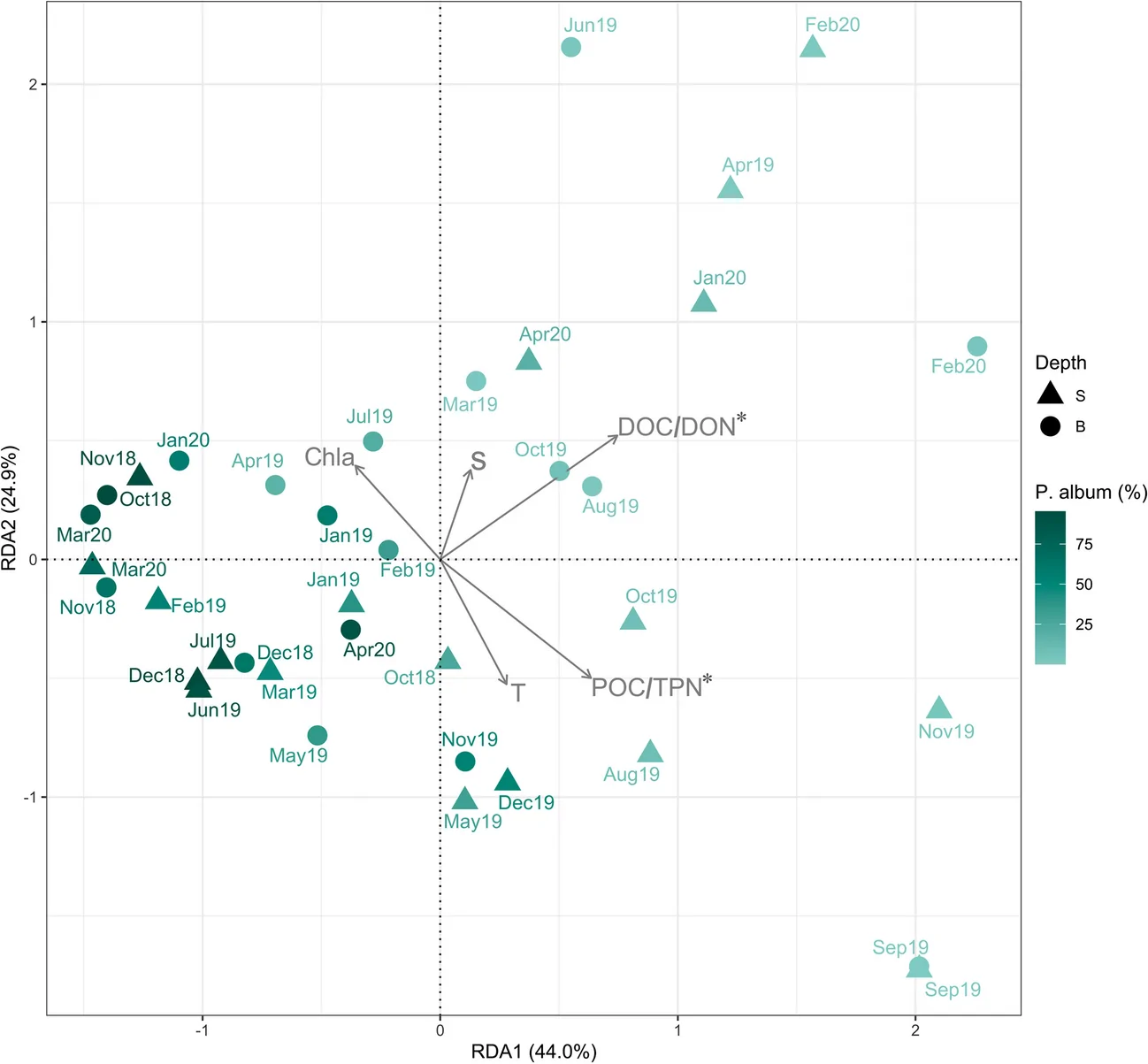
Distance-based redundancy analysis (dbRDA) based on Bray-Curtis dissimilarity in community composition. T = temperature; S = salinity; DOC = dissolved organic carbon; POC = particulate organic carbon; DON = dissolved organic nitrogen; TPN = total particulate nitrogen. Asterisks indicate significant environmental variables

The dbRDA (Fig. 3) showed that DOC/DON and TOC/TPN ratios had significant roles (*p* < 0.001 and *p* < 0.05 respectively) in shaping the fungal communities and in differentiating samples in terms of diverse proportion of *P. album*.

Multivariate analysis did not highlight significant differences between surface and bottom samples in terms of taxonomic composition and environmental drivers.

### Marine Genera

**Fig. 4:**
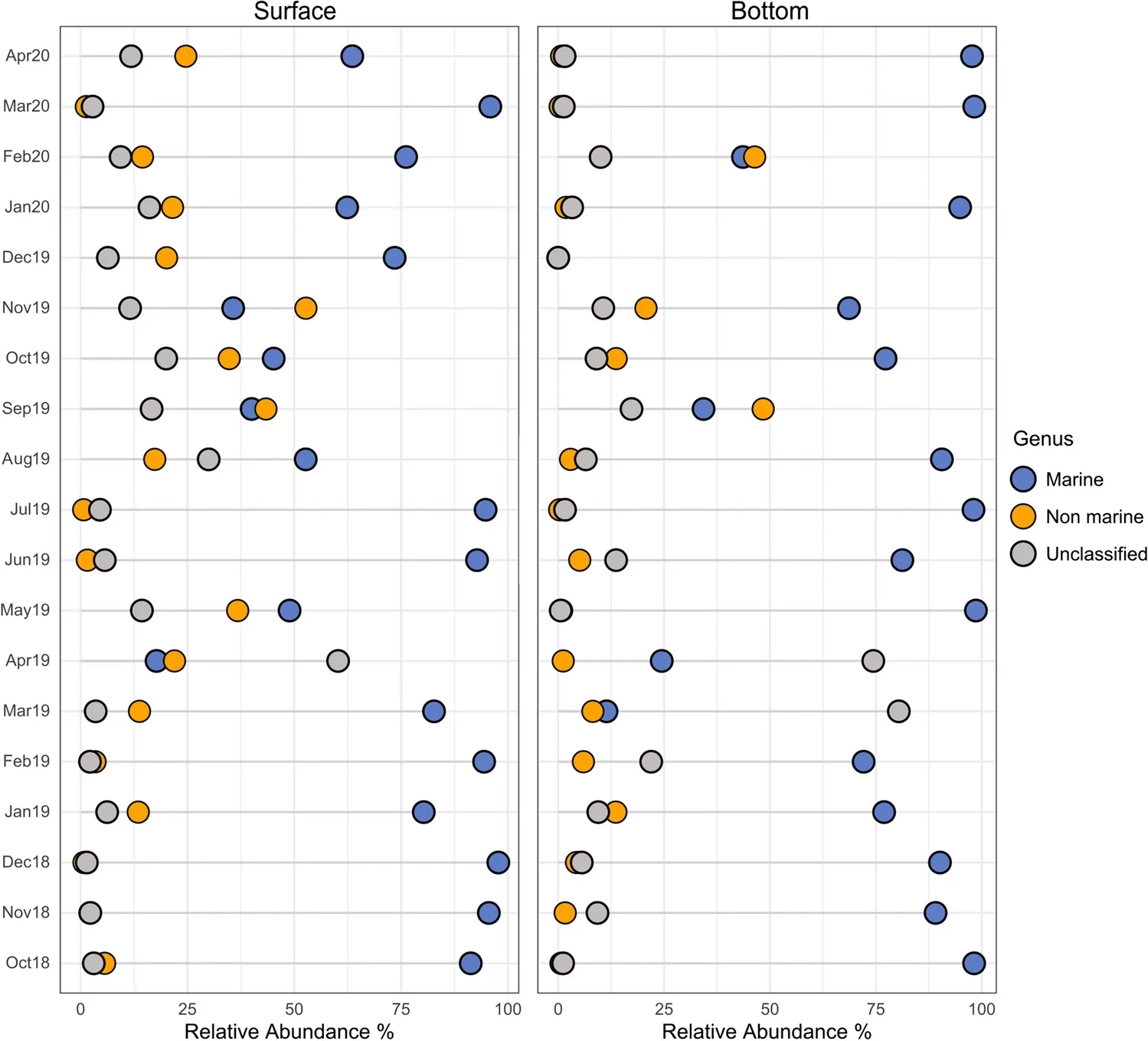
Lollipop chart showing the proportion of marine, non-marine, and unclassified genera for each sample. Only identified genera with an average relative abundance > 0.01% are included

Among the non-rare 227 defined genera (Table S1), 47 were defined as marine, representing 82% of the total dataset in terms of relative abundance. The corresponding OTUs were 309 with an average of 69 ± 33 per sample, corresponding to 72.6 ± 25.8% of the reads on average per sample (Fig. 4).

The marine genera belonged to three phyla: Ascomycota, Basidiomycota, and Basidiobolomycota and included all "core" as well as all the most abundant genera (average > 0.5%) except for *Knufia* and *Peniophora* (Fig. 2). The dbRDA (Fig. S3) performed on this subset highlighted DOC/DON and TOC/TPN ratios as significant drivers (*p* < 0.001 and *p* < 0.05, respectively) of the marine fungal communities and in differentiating samples in terms of different proportion of *P. album*.

### 18S V4 Analysis

For 18S V4, a total of 3,790,777 raw reads were generated for the 19 samples. After primer removal, denoising, and chimera deletion, 2,873,322 reads were retained, with an average of 151,128 ± 50,679 reads and 740 ± 278 ASVs per sample for a total of 7177 ASVs. Most reads belonged to the phyla Dinoflagellata (23.6%), while the proportion of fungal taxa (Kingdom Fungi) was 0.7% (20,906 on 2,873,322 reads and 209 on 7177 ASVs). In only four samples (Mar19_S, Jul19_S, Nov18_S, Oct19_S, Feb19_S), the number of fungal reads was higher than 1000. Unlike for ITS1, reads belonging to Labyrinthulomycetes (Straminipila, 0.6% of the dataset) were detected.

The taxonomy of the samples, presented as interactive Krona charts [37], is available at https://github.com/ElisaBanchi/Fungi_ITS. Four phyla (Ascomycota, Basidiomycota, Chytridiomycota, and Rozellomycota) were detected (Fig. S4). The number of genera detected amounted to 47 (of which 41 were identified with a generic name). The most abundant (average > 0.5%; Fig. S4) genera were *Cystobasidium*, *Gjaerumia*, *Kondoa*, *Malassezia*, *Metschnikowia*, *Nowakowskiella*, *Paramicrosporidium*, *Rhizophydium*, *Rhodotorula*, and *Sakaguchia.*

**Fig. 5:**
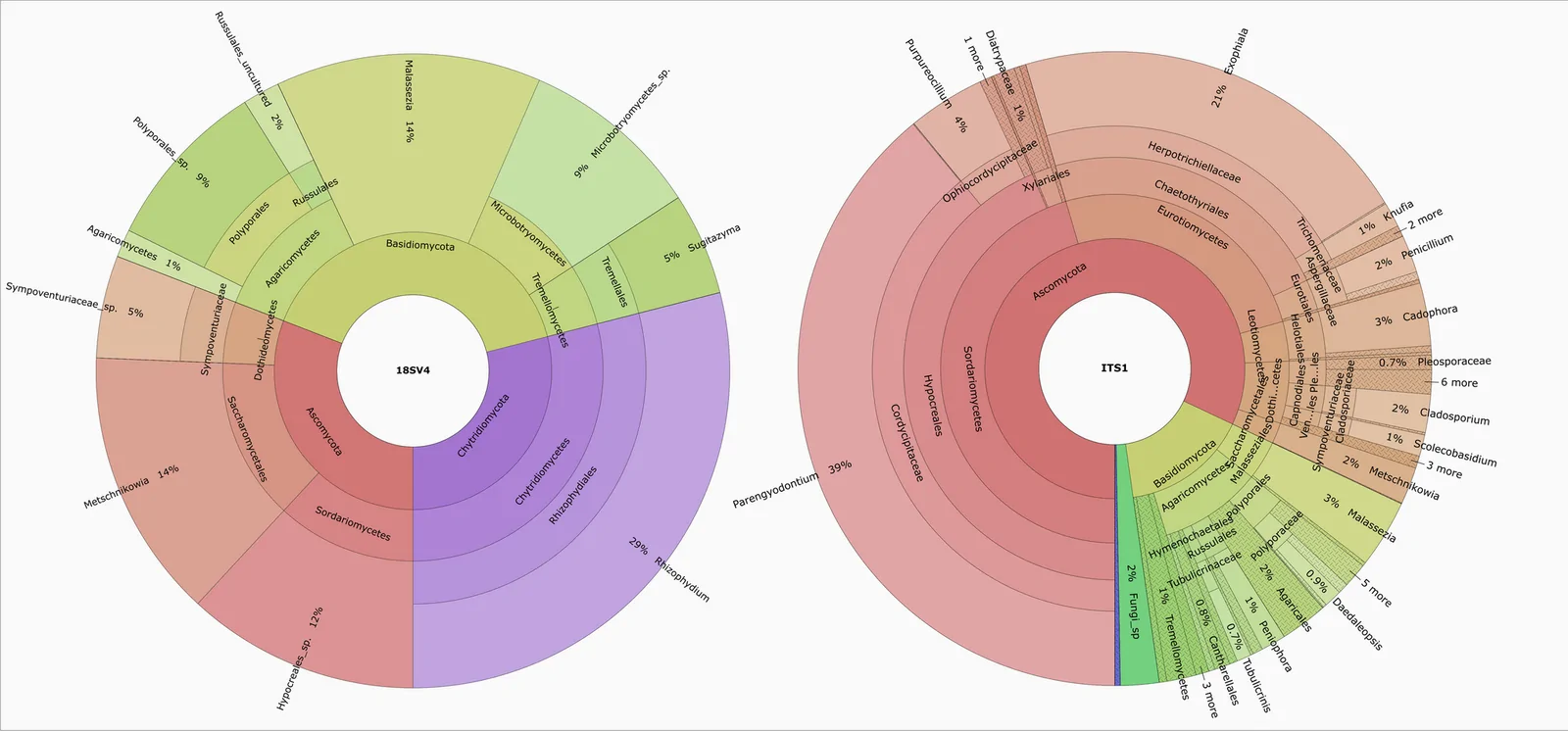
Krona charts showing the taxonomic composition of the C1 surface sample Jan19 using 18S V4 (left panel) and ITS1 (right panel) as barcode

Of the 41 genera identified with 18S V4, 31 were in common with ITS1 (considering only surface samples) (Table S2). Since 18S V4 had a low proportion of fungal reads, the number of taxa detected in each sample was correspondingly low. For example, the number of genera per sample averaged 4.5 ± 4 for 18S V4, while it was 144 ± 88 for ITS1. The different proportion and resolution of the fungal reads obtained with the two barcode genes is reflected in the different taxonomic composition of the same sample. An example of this is shown in Fig. 5 for Jan19_S. Here, the number of OTUs/ASVs was 10 for 18S V4 and 141 for ITS1, of which 4 and 122, respectively, could be assigned at the genus level. In addition to the relative abundances, presence/absence patterns were different: Chytridiomycota, for example, were found with 18S V4 but not with ITS1, while the contrary was true for Rozellomycota.

## Discussion

### A Diverse and Dynamic Mycobiome Shaped by Organic Matter Quality

Overall, the fungal community detected at the LTER station C1 was in line with results from recent DNA metabarcoding surveys in coastal and open waters [7, 15, 17, 29, 38, 39]. In fact, we detected a prevalence of Dikaryotic fungi, Ascomycota, and, in minor proportion, Basidiomycota, in surface and bottom samples (Fig. 2). Chytridiomycota, instead, were abundant in a few samples only. While we could assign most of the detected Ascomycota and Basidiomycota to the genus level, Chytridiomycota showed a lower taxonomic resolution. This also applies to the lower taxonomic levels, with the most common genera such as *Aspergillus*, *Aureobasidium*, *Candida*, *Cladosporium*, *Cryptococcus*, *Malassezia*, *Penicillium*, *Parengyodontium*, and *Rhodotorula* being among the taxa most frequently detected in marine ecosystems [2, 5, 15].

The fungal communities we detected included both yeasts (single-celled: *Exophiala*, *Malassezia*, *Metschnikowia*) and filamentous forms (hyphal: *Acremonium*, *Aspergillus*, *Cladosporium*, *Parengyodontium*, *Penicillium*), reflecting the complexity of this planktonic fraction. Moreover, from an “amplicon sequencing” point of view, the detection of organisms (or portions of them) with mycelial (filamentous, multicellular), unicellular (yeasts), and possibly multinucleate forms, as well as the number, size, and type of spores produced, could represent a bias in different steps of the workflow, including the relative proportion of reads.

A common feature of mycoplankton is its high dynamicity at both the spatial and temporal levels [2, 17], and with respect to other planktonic organisms (e.g., bacterioplankton), it is generally characterized by a patchier distribution, largely dependent on the quality and availability of organic carbon [40]. In fact, both yeast and filamentous fungi can be attached to large particles and can colonize microzones where there is a large availability of organic matter [29, 38]. Moreover, a similar situation can be hypothesized to happen in seawater as described for freshwater streams, where, based on the availability of substrates, fungal mycelia and conidia can show boom-bust cycles [41]. Chrismas et al. [15], in the longest time-series on mycoplankton (17 years), recovered different recurrence patterns: annually (e.g., *Metschnikowia*), persistent (e.g., *Cladosporium*, *Symmetrospora*), occasional (e.g., *Rhodotorula*, *Parengyodontium*), or random (e.g., *Penicillium*). Also, in our survey, the compositional patterns were rather variable and patchy along the sampling period at both depths. Indeed, none of the taxa was steadily present (Fig. 2), rather showing episodic peaks decoupled from the hydrological or biological features of the study area (Fig. 3), differently from what was described for bacterioplankton [42] and phytoplankton [43]. For this reason, we used a hierarchical clustering approach to highlight similarity among samples.

From this analysis, *Parengyodontium album* emerged as a key taxon of the northern Adriatic mycoplankton, as well as the most abundant (37.2 ± 33.0%, Fig. 2) and part of the core mycobiome. *P. album* is a filamentous, chemoorganotrophic species often detected in seawater [15, 39]. It has also been found in sediments and associated with sponges, corals, and crustaceans and regarded as a potential pathogen of marine animals [44]; it is also considered an emerging opportunistic human pathogen due to its proteolytic and keratinolytic activities [45]. Other abundant and part of the core mycobiome taxa of our C1 site such as *Aspergillus*, *Cladosporium*, *Exophiala*, and *Penicillium* (Fig. 2), together with other fungi present in lower abundance such as *Rhodotorula*, *Mortierella*, and *Trichoderma*, are known to be able to degrade recalcitrant and complex molecules including oil, hydrocarbons, and lignin [1, 46]. *Cladosporium* is a cosmopolitan fungal genus commonly detected in both terrestrial [47] and aquatic environments, with halo- and osmotolerant species adapted to marine environments [29]. Moreover, Cunliffe et al. [48] using DNA stable-isotope probing showed that well-represented marine strains of *Cladosporium* can assimilate algal-derived POC. The utilization of these compounds highlighted a saprotrophic functional role of these taxa in processing algal polysaccharides and, as they can potentially be eaten by zooplankton, a link for C transfer to higher trophic levels [48].

In marine ecosystems, in analogy with terrestrial processes, fungi can occur mainly as saprotrophs or symbionts, occupying a wide range of ecological niches [5]. Saprotrophic fungi, especially Dikarya, can secrete (exo-)hydrolytic enzymes to process labile as well as refractory organic molecules [38, 40]. These include recalcitrant materials (with high C:N ratios) that are more difficult for bacterioplankton to utilize, suggesting a complementary role for these two fractions [17]. Fungi can play an important role in marine food webs by building complex biotrophic interactions with phytoplankton and zooplankton (such as parasitism, predation, grazing, and pathogenicity) and can shape carbon fluxes within planktonic food webs *via* the mycoloop [6, 38, 40]. Besides the conceptual importance of mycoplankton, the main drivers of its compositional and diversity patterns, as well as its temporal and spatial dynamics, remain largely unclear [7, 17]. This is primarily due to the limited number of studies that have investigated these organisms, which are scattered both temporally and spatially (e.g., coastal vs open ocean) and therefore span different ecosystems and environmental gradients. In addition, the different barcode targets and taxonomic resolutions gained or considered make it difficult to integrate and compare the results of the different studies. In general, fungal communities are the result of the interplay of multiple environmental factors [17]. Breyer and Baltar [7] in their meta-analysis listed potential influencing drivers: (*i*) abiotic factors (such as temperature, depth, pH, salinity), (*ii*) the availability of major elements (C, N, P), and (*iii*) biotic factors (abundance and composition of phytoplankton and zooplankton).

In this study, no consistent role of chlorophyll *a* concentration was observed (Fig. 3), evidencing that the relationship between fungi and microalgae is mainly sporadic. On the other hand, dissolved (DOC/DON) and particulate (TOC/TPN) organic carbon and nitrogen ratios were significant drivers of the fungal communities (Fig. 3). In another study in the Mediterranean (Ligurian Sea), Celussi et al. [20] hypothesized that a higher presence of Fungi in samples with a higher C:N ratio could explain the observed higher rates of organic matter degradation. Similarly, Bochdansky et al. [49] found a high fungi-to-prokaryotes ratio, in terms of biomass, associated with deep-sea marine snow, rich in refractory organic compounds [50].

Noteworthy in our dataset, different mycoplankton assemblages were associated with either dissolved or particulate C:N ratio. For instance, in the samples characterized by higher dissolved C:N ratio, the yeast-forming genera *Kondoa* and *Malassezia* were more abundant, while the filamentous and cosmopolitan *Cladosporium* and *Alternaria* genera were more present in samples with higher particulate C:N ratio, suggesting that different fungal consortia may rely on these different fractions of recalcitrant organic matter. While the role of marine fungi in the processing of dissolved refractory compounds is difficult to quantify, there is evidence of their capability to degrade dissolved humic compounds (high C:N) through multiple degradation pathways [3]. Taken together, these findings strengthen the idea that in temperate marine waters, mycoplankton may play a complementary role alongside bacterioplankton in organic matter cycling, possibly breaking down complex organic compounds and thus increasing their bioavailability, boosting the microbial loop.

### The Coastal Environment as a Sink for Terrestrial Fungi

Although this work focuses mainly on marine taxa, the identification and exploration of the terrestrial fraction are interesting and allow for a more comprehensive assessment of the biodiversity of the studied area, the influence of land use, and the evaluation of land-sea exchanges (e.g., input of organic matter and nutrients).

The occurrence of terrestrial fungi (Dikarya) in coastal and oceanic habitats has been linked to their reproductive success and airborne dispersal. Their spores can be transported over long distances and at high speed [33]; therefore, fungi living in woods or rocks can easily be transported in water. In this respect, coastal waters act as a sink for land-derived fungi [51], increasing mycobiome diversity. For example, Chrismas et al. [15] detected different genera associated with forests as well as a lichen-forming genus (*Lichina*) present along the coast near their sampling site. In our dataset, the most abundant non-marine genera were *Knufia* and *Peniophora*. *Knufia* is an oligotrophic and halotolerant, rock-inhabiting Ascomycota, a persistent dweller of natural and anthropogenic extreme habitats [52]. Even if the presence of fungi of terrestrial origin in seawater should be considered random, which is also related to higher or lower dispersal capabilities, we cannot exclude the possibility that halophilic taxa could survive in this environment. *Peniophora*, a filamentous Basidiomycota, is a widespread genus that grows on decaying wood and plant decay material [53]: it is likely that the amplified reads derived from mycelia on wood and truck pieces have entered the sea either transported by winds, rivers, floods, or tides.

### Taxonomic Resolution and Detection of Early Divergent Fungi: ITS and 18S

The choice of a target region for DNA metabarcoding studies is a critical step as it influences the amplification procedures (e.g., amplicon length, number of cell/copies) as well as the bioinformatic pipelines and taxonomic assignment (e.g., reference databases). For fungal communities, a consensus has been found on the choice of internal transcribed spacer (ITS) [54], with studies targeting either subregions 1 (ITS1) or 2 (ITS2) [7] or even aiming at sequencing the whole ITS region through the latest third-generation sequencing technologies (e.g., Oxford Nanopore, Pacific Biosciences).

In this study, we identified different proportions of fungal reads obtained with the two barcodes (31% for ITS1 and 0.7% for 18S V4), resulting in a tenfold difference in the number of identified genera (Fig. 5, Table S2). If the 18S rRNA gene has lower variation and thus lower taxonomic accuracy and resolution than the ITS region [14], which also emerged in our study (e.g., for Hypocreales), the use of the ITS as a marker may introduce biases and lack the detection of some early-diverging lineages. For example, in 18S V4, we found that the proportion of Chytridiomycota was higher compared to ITS1, but still largely undefined at the lower taxonomic levels. However, some genera were identified, including the marine *Olpidium* (comprising mainly obligate endoparasites of algae, plants, fungi, and animals [55]) and *Rhizophydium* (comprising diatom parasites [56]). The assignment of Chytridiomycota reads to lower taxonomic levels is challenging due to the lack of reference sequences in public databases including UNITE [27] and to their high genetic divergence [56]. As a result, the contribution of this marine basal group to the transfer of organic matter and nutrients within food webs is likely underestimated [5]. Moreover, it is worth noticing that the pattern of Chytridiomycota abundance, which commonly depends on host-parasite biotic interactions with phytoplankton, is not always evident [57]. Indeed, we could not establish a clear relationship between chytrids and phytoplankton, possibly because the Chytridiomycota we detected could be associated with saprotrophic or parasitic organisms besides phytoplankton, as already suggested by Banos et al. [38]. We could also speculate that, if an event (e.g., a diatom bloom) occurred, it was too short to be captured by the sampling frequency we used for this study.

Overall, our study confirmed that ITS has a high taxonomic resolution within mycoplankton, but further improvement of reference databases is needed to better identify marine taxa, especially Chytridiomycota. If 18S data obtained with universal eukaryotic primers are available, screening of fungal reads may provide complementary information on early divergent fungal lineages. However, we suggest approaching these data in terms of presence/absence rather than in terms of relative abundance, to exploit the detection power of the barcode while at the same time partially accounting for the biases due to the low number of fungal reads.

## Conclusion

This is, to the best of our knowledge, the first study targeting mycoplankton in the Adriatic Sea. The fungal communities in the C1 LTER coastal site were very diverse and dynamic, with Dikarya and sporadically Chytridiomycota as prevalent taxa. The quality and availability of organic matter likely play an important role in the distribution of fungi, and *Parengyodontium album* was identified as a key taxon in the mycoplankton of the northern Adriatic, but further efforts are needed to clarify its functional role in the coastal environment.

New insights into the characterization of mycoplankton could be gained by using long-reads DNA metabarcoding covering the 18S-ITS-28S region in conjunction with the isolation and cultivation of marine fungal strains potentially belonging to representative species and to early divergent lineages.

Improving the spatial and taxonomic resolution of the marine fungal community will help to clarify the role of mycoplankton in marine food webs and biogeochemical cycles and to develop an integrated picture of the structure and functions of the coastal environment.

## Electronic Supplementary Material

Below is the link to the electronic supplementary material.

Supplementary Material 1

Supplementary Material 2

## Data Availability

The nucleotide sequence data reported are available in the Sequence Reads Archive (SRA) at NCBI under the accession numbers PRJNA1061483.
