# Supplementary material for: Marine Fungal Diversity and Dynamics in the Gulf of Trieste (Northern Adriatic Sea)
Source: Microb Ecol. 2024 May 29;87(1):78. doi: 10.1007/s00248-024-02394-z (PMC11133070; doi:10.1007/s00248-024-02394-z)
Supplement: Supplementary file 2 — Supplementary Material 2 [file 248_2024_2394_MOESM2_ESM.docx]

**SUPPLEMENTARY DATA**

**Marine fungal diversity and dynamics in the Gulf of Trieste (northern Adriatic Sea)**

Elisa Banchi^1,2*^, Vincenzo Manna^1,2^, Lucia Muggia^3^, Mauro Celussi^1,2^

^1^National Institute of Oceanography and Applied Geophysics – OGS, Trieste, Italy

^2^NBFC, National Biodiversity Future Center, Palermo, Italy

^3^Department of Life Sciences, University of Trieste, Trieste, Italy

***Corresponding author:**

Elisa Banchi [ebanchi@ogs.it](mailto:ebanchi@ogs.it)

National Institute of Oceanography and Applied Geophysics - OGS

via Piccard 54 34151 Trieste, Italy

Tel +39 0402140721; Fax +39 0402249770

SUPPLEMENTARY RESULTS

Dissolved organic carbon (DOC) concentration showed a marked seasonal dynamic, with winter minima and summer maxima, ranging between 72.26 and 143.87 µmol L^-1^ (Fig. S1). In surface waters, spikes in DOC concentration occurred in April 2019 and February 2020, while the trend was smoother in bottom samples (Fig. S1).

Particulate organic carbon (POC) concentration shared similar winter dynamics with minima between January and February (8.55±1.26 µmol L^-1^ on average; Fig. S1). Two distinct maxima were evident in surface samples, in June and October 2019 (31.20 and 26.29 µmol L^-1^, respectively; Fig. S1), while in the bottom layer a single peak occurred in June 2019 (25.45 µmol L^-1^; Fig. S1).

C/N calculated for the dissolved organic matter pool were overall similar in the surface and bottom layers (14.62±3.90 and 14.46±2.90, respectively). In surface samples an oscillating trend was evident, with minima in November 2018 (6.90; Fig. S1) and maxima in March 2020 (16.78; Fig. S1). Bottom dynamics were steadier, with lower values in autumn and winter, increasing during summer. The March 2020 peak was measured also in the bottom layer of the water column (Fig. S1).

On average, C/N in the particulate pool was very similar between surface and bottom samples (7.75±1.56 and 7.74±1.75, respectively; Fig. S1). At both depths, higher values were found in December 2018 and September 2019. Noteworthy, while a minimum was evident in February 2020 in surface samples (5.12; Fig. S1), bottom values displayed a maximum (11.96; Fig. S1).

SUPPLEMENTARY FIGURES

**
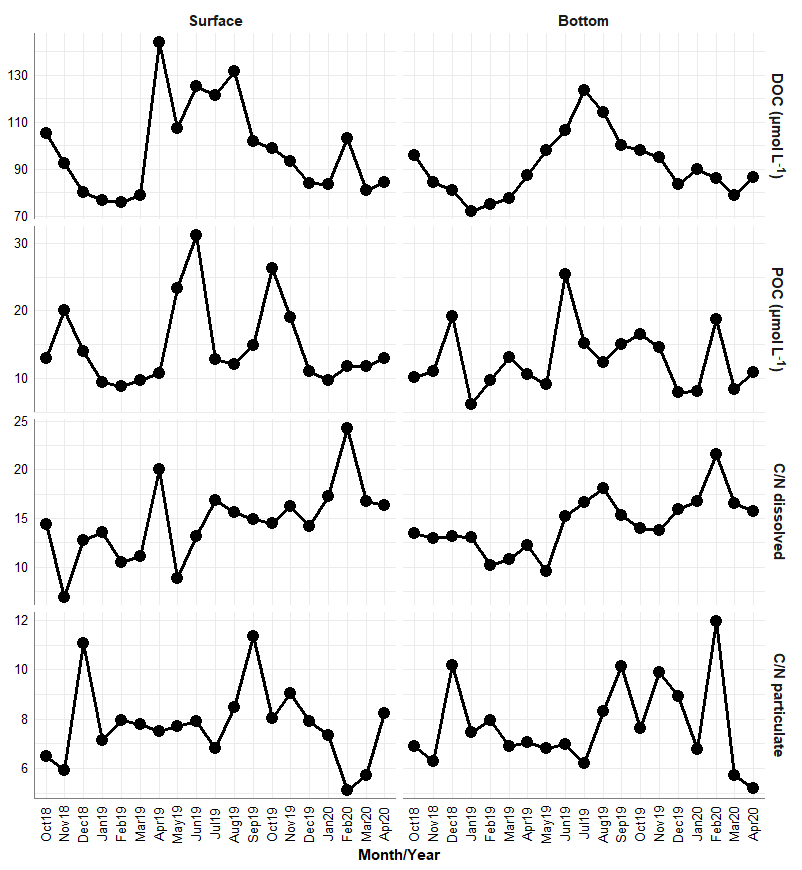
**

**Supplementary Fig. S1** Trends of Dissolved organic carbon (DOC), Particulate organic carbon (POC), C/N of the dissolved and of the particulate pool in C1 surface and bottom samples


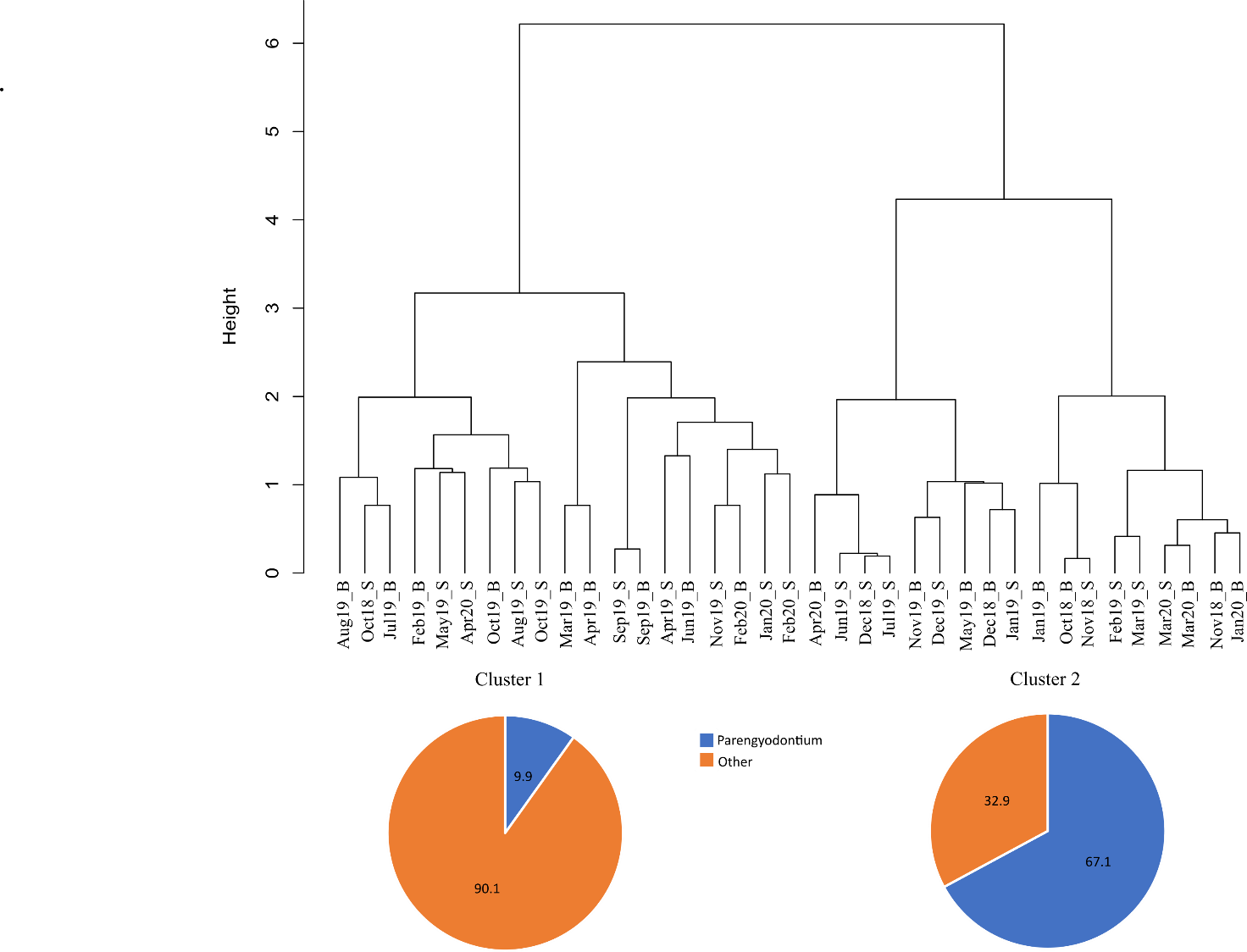


**Supplementary Fig. S2** Hierarchical cluster dendrogram (Ward.D2 method) based on fungal OTUs. The pie charts show the average proportion of *P. album* in the two clusters


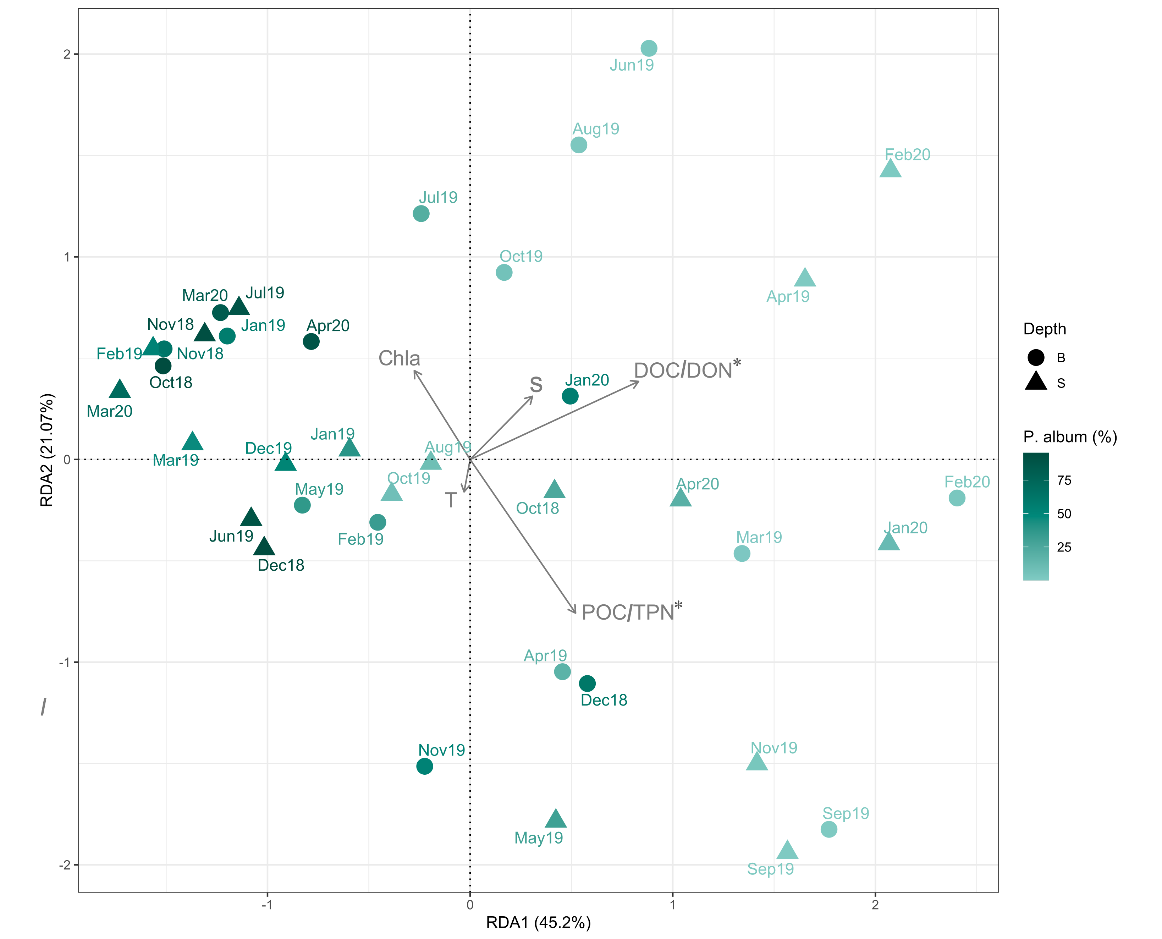


**Fig. S3** Distance-based redundancy analysis (dbRDA) based on Bray-Curtis dissimilarity in community composition taking into account the OTUs of marine genera. T = temperature; S = salinity; DOC = dissolved organic carbon; POC = particulate organic carbon; DON = dissolved organic nitrogen; TPN = total particulate nitrogen. Asterisks indicate significant environmental variables


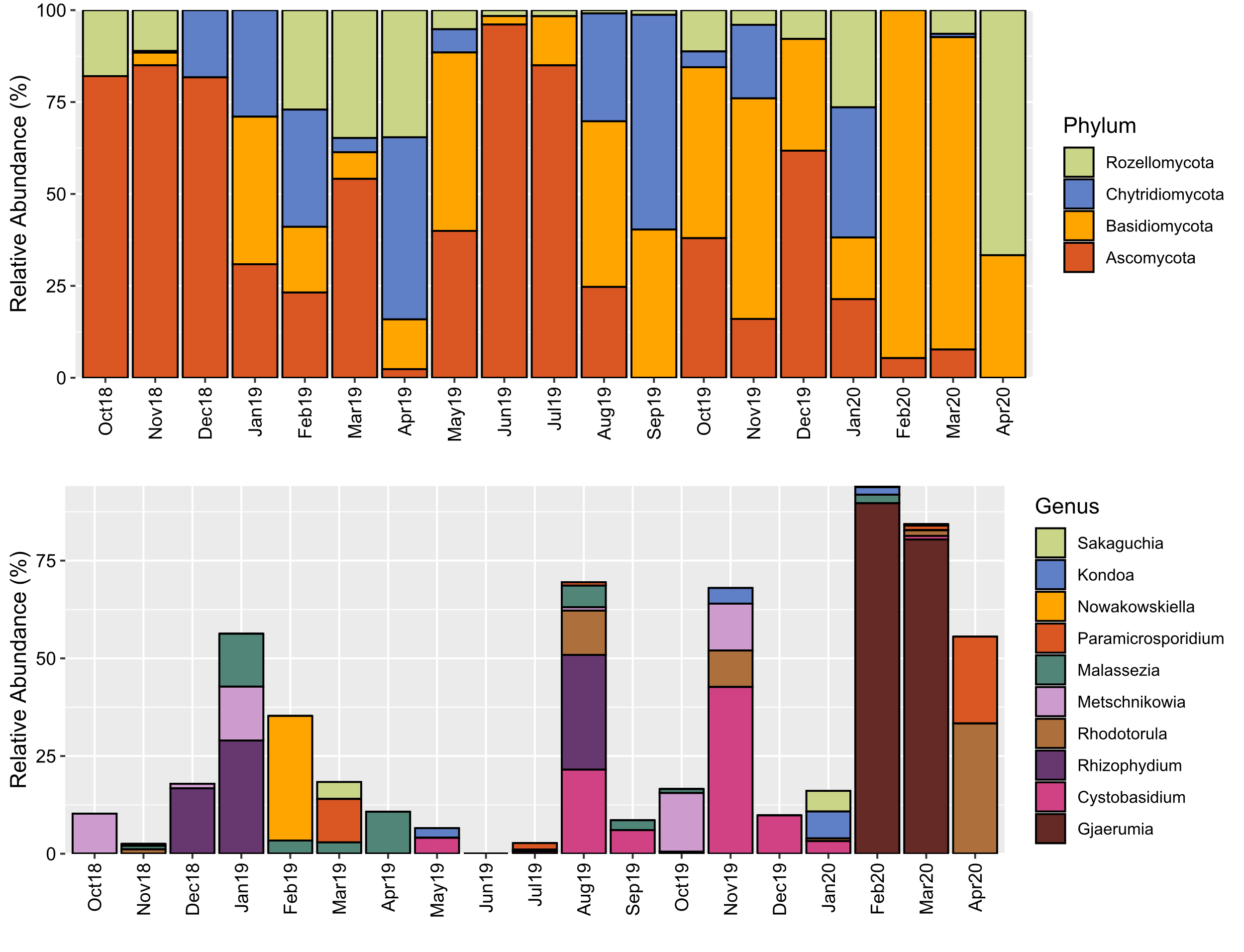


**Supplementary Fig. S4** Taxonomic composition of marine fungi at the phylum (upper panel) and genus (lower panel) level assigned with 18S V4. Genera with an average abundance > 0.5% are shown

SUPPLEMENTARY TABLES

**Supplementary Table S1**

Fungal genera detected with ITS1 DNA metabarcoding. For each, phylum (A = Ascomycota; Ba = Basidiobolomycota; B = Basidiomycota; C = Chytridiomycota; F = Fungi sp.; M = Mortierellomycota; R = Rozellomycota), average and standard deviation (SD) across the dataset, and feature (Marine, non Marine, Rare, Not defined) is reported. For marine genera, the trophic mode based in FUNguild (Nguyen et al. 2016) is showed (NA: not assigned)

| **Phylum** | **Genus** | **Average** | **SD** | **Comment** |
| --- | --- | --- | --- | --- |
| A | *Parengyodontium* | 37.725037 | 33.061609 | Marine (Pathotroph-Saprotroph) |
| A | *Exophiala* | 10.640300 | 12.005630 | Marine (Pathotroph) |
| A | *Purpureocillium* | 4.033007 | 10.788288 | Marine (Pathotroph) |
| A | *Cadophora* | 2.548581 | 5.154201 | Marine (Symbiotroph) |
| A | *Scolecobasidium* | 2.548136 | 6.004810 | Marine (Saprotroph) |
| B | *Kondoa* | 2.229955 | 8.688730 | Marine (NA) |
| A | *Cladosporium* | 2.214110 | 3.376635 | Marine (Saprotroph-Symbiotroph) |
| B | *Malassezia* | 2.092965 | 4.541200 | Marine (Pathotroph-Saprotroph) |
| A | *Metschnikowia* | 1.696932 | 7.014051 | Marine (NA) |
| A | *Alternaria* | 1.010311 | 1.981049 | Marine (Pathotroph-Saprotroph-Symbiotroph) |
| A | *Ochroconis* | 0.880986 | 3.787929 | Marine (Saprotroph) |
| B | *Cystobasidium* | 0.708708 | 1.479268 | Marine (Pathotroph) |
| B | *Rhodotorula* | 0.476004 | 0.978714 | Marine (Pathotroph-Saprotroph) |
| A | *Aspergillus* | 0.454331 | 0.589474 | Marine (Pathotroph) |
| B | *Sakaguchia* | 0.449828 | 1.167293 | Marine (Saprotroph) |
| A | *Penicillium* | 0.423923 | 0.398572 | Marine (Saprotroph) |
| A | *Aureobasidium* | 0.324693 | 0.400634 | Marine (Pathotroph-Saprotroph-Symbiotroph) |
| A | *Acremonium* | 0.313450 | 0.857533 | Marine (Pathotroph-Saprotroph-Symbiotroph) |
| B | *Symmetrospora* | 0.300974 | 0.853649 | Marine (NA) |
| B | *Schizophyllum* | 0.204580 | 0.718258 | Marine (Saprotroph) |
| B | *Filobasidium* | 0.145435 | 0.213978 | Marine (Saprotroph) |
| A | *Fusarium* | 0.144941 | 0.209183 | Marine (Pathotroph-Saprotroph-Symbiotroph) |
| A | *Trichoderma* | 0.116615 | 0.247623 | Marine (Pathotroph-Saprotroph-Symbiotroph) |
| B | *Cystofilobasidium* | 0.094939 | 0.207678 | Marine (Saprotroph) |
| A | *Phaeosphaeria* | 0.083144 | 0.161108 | Marine (Saprotroph) |
| B | *Trechispora* | 0.081194 | 0.136069 | Marine (Saprotroph) |
| A | *Debaryomyces* | 0.055923 | 0.116188 | Marine (Saprotroph) |
| A | *Eutypa* | 0.053391 | 0.106153 | Marine (Pathotroph) |
| A | *Eutypella* | 0.051285 | 0.124785 | Marine (Pathotroph) |
| B | *Sporobolomyces* | 0.050898 | 0.162874 | Marine (Pathotroph-Saprotroph) |
| A | *Plectosphaerella* | 0.046805 | 0.181220 | Marine (Pathotroph-Symbiotroph) |
| B | *Ganoderma* | 0.042560 | 0.083810 | Marine (Pathotroph-Saprotroph) |
| A | *Coniosporium* | 0.037744 | 0.077536 | Marine (Pathotroph) |
| A | *Candida* | 0.035899 | 0.066777 | Marine (Pathotroph-Saprotroph-Symbiotroph) |
| A | *Neosetophoma* | 0.033484 | 0.065908 | Marine (Saprotroph) |
| A | *Paraphoma* | 0.032801 | 0.097073 | Marine (Pathotroph) |
| A | *Stemphylium* | 0.032641 | 0.070587 | Marine (Pathotroph-Saprotroph) |
| A | *Talaromyces* | 0.026965 | 0.075371 | Marine (Saprotroph) |
| A | *Periconia* | 0.026962 | 0.082401 | Marine (Pathotroph-Saprotroph-Symbiotroph) |
| B | *Erythrobasidium* | 0.026621 | 0.055205 | Marine (NA) |
| A | *Phaeoisaria* | 0.025303 | 0.070019 | Marine (Saprotroph) |
| A | *Pseudorobillarda* | 0.019821 | 0.064264 | Marine (Pathotroph) |
| A | *Cytospora* | 0.019752 | 0.050164 | Marine (Pathotroph-Saprotroph-Symbiotroph) |
| A | *Sarocladium* | 0.017948 | 0.064519 | Marine (Saprotroph) |
| Ba | *Basidiobolus* | 0.013204 | 0.080317 | Marine (Pathotroph-Saprotroph-Symbiotroph) |
| B | *Sterigmatomyces* | 0.013186 | 0.042642 | Marine (NA) |
| A | *Cryptovalsa* | 0.012635 | 0.028575 | Marine (NA) |
| A | *Knufia* | 4.083713 | 8.181212 | non Marine |
| B | *Peniophora* | 0.817999 | 1.630803 | non Marine |
| A | *Peroneutypa* | 0.361719 | 0.855927 | non Marine |
| B | *Pseudozyma* | 0.314076 | 1.064282 | non Marine |
| B | *Stereum* | 0.292088 | 0.715015 | non Marine |
| B | *Trametes* | 0.257646 | 0.454532 | non Marine |
| B | *Rhodosporidiobolus* | 0.251944 | 1.395590 | non Marine |
| B | *Armillaria* | 0.237093 | 0.813072 | non Marine |
| B | *Phlebia* | 0.212180 | 0.527417 | non Marine |
| B | *Hyphodermella* | 0.184254 | 0.458334 | non Marine |
| A | *Mollisia* | 0.180418 | 0.800289 | non Marine |
| B | *Vuilleminia* | 0.171804 | 0.363113 | non Marine |
| A | *Diatrype* | 0.167817 | 0.322898 | non Marine |
| B | *Sidera* | 0.140985 | 0.348144 | non Marine |
| B | *Bjerkandera* | 0.135888 | 0.251697 | non Marine |
| B | *Strobilurus* | 0.135635 | 0.428416 | non Marine |
| A | *Didymella* | 0.131241 | 0.278560 | non Marine |
| B | *Curvibasidium* | 0.130714 | 0.512382 | non Marine |
| B | *Gymnopus* | 0.126020 | 0.234002 | non Marine |
| A | *Gibberella* | 0.123670 | 0.311299 | non Marine |
| A | *Neoascochyta* | 0.120574 | 0.514606 | non Marine |
| A | *Nigrospora* | 0.119905 | 0.331080 | non Marine |
| B | *Steccherinum* | 0.109910 | 0.273574 | non Marine |
| B | *Mycena* | 0.108061 | 0.235417 | non Marine |
| A | *Pseudopithomyces* | 0.105046 | 0.398124 | non Marine |
| B | *Burgoa* | 0.104328 | 0.370084 | non Marine |
| B | *Exidia* | 0.096974 | 0.353317 | non Marine |
| B | *Fuscoporia* | 0.095915 | 0.178976 | non Marine |
| B | *Clitocybe* | 0.088467 | 0.228896 | non Marine |
| B | *Sistotrema* | 0.087117 | 0.125563 | non Marine |
| B | *Daedaleopsis* | 0.083716 | 0.216691 | non Marine |
| B | *Coprinellus* | 0.082552 | 0.133153 | non Marine |
| B | *Cabalodontia* | 0.079289 | 0.140736 | non Marine |
| A | *Ramularia* | 0.079229 | 0.181151 | non Marine |
| A | *Lecanicillium* | 0.078830 | 0.127374 | non Marine |
| B | *Cutaneotrichosporon* | 0.073241 | 0.327104 | non Marine |
| B | *Naganishia* | 0.072773 | 0.244318 | non Marine |
| A | *Iodophanus* | 0.068144 | 0.413690 | non Marine |
| A | *Curvularia* | 0.063069 | 0.100781 | non Marine |
| A | *Hortaea* | 0.061313 | 0.144050 | non Marine |
| B | *Hypholoma* | 0.060756 | 0.151935 | non Marine |
| B | *Ceriporia* | 0.060546 | 0.135248 | non Marine |
| B | *Radulomyces* | 0.060147 | 0.141324 | non Marine |
| A | *Xenodidymella* | 0.060078 | 0.135391 | non Marine |
| B | *Tausonia* | 0.056549 | 0.209971 | non Marine |
| A | *Taphrina* | 0.053625 | 0.100950 | non Marine |
| A | *Neocatenulostroma* | 0.053053 | 0.101447 | non Marine |
| A | *Botrytis* | 0.050542 | 0.118773 | non Marine |
| A | *Leptospora* | 0.050142 | 0.145035 | non Marine |
| B | *Vishniacozyma* | 0.049733 | 0.096201 | non Marine |
| A | *Bartalinia* | 0.049474 | 0.158294 | non Marine |
| A | *Neophaeococcomyces* | 0.047874 | 0.119136 | non Marine |
| B | *Porostereum* | 0.047266 | 0.137630 | non Marine |
| B | *Wallemia* | 0.046793 | 0.100701 | non Marine |
| A | *Pseudoophiobolus* | 0.046671 | 0.150313 | non Marine |
| A | *Articulospora* | 0.042246 | 0.127670 | non Marine |
| A | *Phaeococcomyces* | 0.041427 | 0.124144 | non Marine |
| B | *Skeletocutis* | 0.038829 | 0.103554 | non Marine |
| A | *Candelaria* | 0.038647 | 0.235080 | non Marine |
| A | *Blumeria* | 0.038646 | 0.136920 | non Marine |
| B | *Xenasmatella* | 0.038490 | 0.073267 | non Marine |
| A | *Flavoplaca* | 0.037655 | 0.155331 | non Marine |
| A | *Sclerostagonospora* | 0.037309 | 0.087047 | non Marine |
| B | *Piskurozyma* | 0.036988 | 0.223373 | non Marine |
| B | *Auricularia* | 0.036580 | 0.087979 | non Marine |
| B | *Cylindrobasidium* | 0.036326 | 0.147126 | non Marine |
| B | *Phanerochaete* | 0.036276 | 0.091065 | non Marine |
| A | *Neocucurbitaria* | 0.036054 | 0.081087 | non Marine |
| B | *Amyloporia* | 0.035622 | 0.090942 | non Marine |
| B | *Erratomyces* | 0.035344 | 0.210575 | non Marine |
| B | *Baeospora* | 0.034117 | 0.087561 | non Marine |
| B | *Lycoperdon* | 0.033876 | 0.068506 | non Marine |
| B | *Gloeoporus* | 0.033443 | 0.111687 | non Marine |
| B | *Genolevuria* | 0.033413 | 0.064892 | non Marine |
| A | *Septoria* | 0.031683 | 0.076952 | non Marine |
| B | *Tubulicrinis* | 0.031049 | 0.122322 | non Marine |
| A | *Erysiphe* | 0.030496 | 0.078213 | non Marine |
| B | *Rhodocollybia* | 0.030426 | 0.054859 | non Marine |
| A | *Paragibellulopsis* | 0.029329 | 0.178402 | non Marine |
| A | *Didymocyrtis* | 0.028884 | 0.081709 | non Marine |
| B | *Ustilago* | 0.028792 | 0.047708 | non Marine |
| B | *Peniophorella* | 0.028784 | 0.053542 | non Marine |
| A | *Ophiognomonia* | 0.028498 | 0.143463 | non Marine |
| A | *Tricellula* | 0.028463 | 0.099849 | non Marine |
| B | *Rigidoporus* | 0.028336 | 0.172360 | non Marine |
| A | *Cylindrium* | 0.028271 | 0.149741 | non Marine |
| B | *Resupinatus* | 0.027944 | 0.076600 | non Marine |
| B | *Cortinarius* | 0.027759 | 0.101771 | non Marine |
| B | *Psathyrella* | 0.027538 | 0.096482 | non Marine |
| B | *Lepista* | 0.027502 | 0.052670 | non Marine |
| A | *Thelebolus* | 0.027284 | 0.141364 | non Marine |
| A | *Kalmusia* | 0.026693 | 0.089640 | non Marine |
| A | *Epicoccum* | 0.026168 | 0.063442 | non Marine |
| B | *Scopuloides* | 0.026098 | 0.077803 | non Marine |
| A | *Naevala* | 0.025815 | 0.134099 | non Marine |
| A | *Neovaginatispora* | 0.025242 | 0.043690 | non Marine |
| A | *Plectania* | 0.024986 | 0.081333 | non Marine |
| B | *Itersonilia* | 0.024594 | 0.066558 | non Marine |
| A | *Emericellopsis* | 0.023836 | 0.095195 | non Marine |
| B | *Apiotrichum* | 0.023775 | 0.106718 | non Marine |
| A | *Paraconiothyrium* | 0.023430 | 0.045986 | non Marine |
| B | *Hyphodontia* | 0.022786 | 0.042694 | non Marine |
| A | *Coniochaeta* | 0.022699 | 0.081061 | non Marine |
| B | *Amyloxenasma* | 0.022676 | 0.050017 | non Marine |
| A | *Cyphellophora* | 0.022410 | 0.068886 | non Marine |
| A | *Stagonosporopsis* | 0.021521 | 0.043882 | non Marine |
| B | *Dioszegia* | 0.021266 | 0.083916 | non Marine |
| B | *Heterobasidion* | 0.021074 | 0.053881 | non Marine |
| B | *Tricholoma* | 0.021023 | 0.058848 | non Marine |
| A | *Paraophiobolus* | 0.021008 | 0.093513 | non Marine |
| A | *Chaetomium* | 0.020762 | 0.123067 | non Marine |
| B | *Byssomerulius* | 0.020295 | 0.043525 | non Marine |
| A | *Phaeosphaeriopsis* | 0.020272 | 0.045666 | non Marine |
| A | *Phialocephala* | 0.020249 | 0.044668 | non Marine |
| A | *Incrucipulum* | 0.020238 | 0.088785 | non Marine |
| A | *Endosporium* | 0.019734 | 0.086765 | non Marine |
| A | *Myrothecium* | 0.019184 | 0.042617 | non Marine |
| B | *Ceriporiopsis* | 0.019074 | 0.050621 | non Marine |
| B | *Amphinema* | 0.019060 | 0.046274 | non Marine |
| A | *Salinomyces* | 0.018764 | 0.056051 | non Marine |
| A | *Fusicolla* | 0.018615 | 0.044057 | non Marine |
| A | *Lapidomyces* | 0.018547 | 0.085193 | non Marine |
| B | *Sertulicium* | 0.018537 | 0.055253 | non Marine |
| B | *Xylodon* | 0.018113 | 0.041155 | non Marine |
| B | *Fonsecazyma* | 0.018005 | 0.062006 | non Marine |
| B | *Coriolopsis* | 0.017854 | 0.041590 | non Marine |
| A | *Parastagonospora* | 0.017839 | 0.044844 | non Marine |
| B | *Ceratobasidium* | 0.017529 | 0.070037 | non Marine |
| A | *Acrodontium* | 0.017368 | 0.069865 | non Marine |
| C | *Clydaea* | 0.017102 | 0.084121 | non Marine |
| B | *Cryptomarasmius* | 0.017018 | 0.038574 | non Marine |
| A | *Pyrenochaetopsis* | 0.016894 | 0.048658 | non Marine |
| A | *Podosphaera* | 0.016790 | 0.102131 | non Marine |
| B | *Effuseotrichosporon* | 0.016142 | 0.077406 | non Marine |
| A | *Teichospora* | 0.016122 | 0.072338 | non Marine |
| A | *Angustimassarina* | 0.016106 | 0.039032 | non Marine |
| B | *Cryptococcus* | 0.016040 | 0.058556 | non Marine |
| A | *Sphaerulina* | 0.015543 | 0.046872 | non Marine |
| B | *Buckleyzyma* | 0.015358 | 0.033352 | non Marine |
| B | *Lenzites* | 0.015320 | 0.054470 | non Marine |
| B | *Botryobasidium* | 0.015259 | 0.048949 | non Marine |
| B | *Bullera* | 0.015235 | 0.045459 | non Marine |
| A | *Cryptostroma* | 0.015201 | 0.071393 | non Marine |
| A | *Heterotruncatella* | 0.015097 | 0.048049 | non Marine |
| A | *Xanthocarpia* | 0.015056 | 0.071849 | non Marine |
| A | *Phaeoacremonium* | 0.014936 | 0.036033 | non Marine |
| A | *Neophaeotheca* | 0.014686 | 0.056323 | non Marine |
| A | *Zalaria* | 0.014671 | 0.058652 | non Marine |
| B | *Cyanosporus* | 0.014191 | 0.036979 | non Marine |
| B | *Hydnum* | 0.013994 | 0.040245 | non Marine |
| C | *Betamyces* | 0.013912 | 0.038092 | non Marine |
| A | *Torula* | 0.013462 | 0.044115 | non Marine |
| A | *Paraphaeosphaeria* | 0.012806 | 0.028966 | non Marine |
| A | *Ochrocladosporium* | 0.012757 | 0.022387 | non Marine |
| B | *Amylostereum* | 0.012713 | 0.069965 | non Marine |
| A | *Perusta* | 0.012653 | 0.056354 | non Marine |
| B | *Exidiopsis* | 0.012484 | 0.031021 | non Marine |
| B | *Sistotremastrum* | 0.012201 | 0.043816 | non Marine |
| A | *Phomatospora* | 0.012054 | 0.035645 | non Marine |
| B | *Gloeocystidiellum* | 0.012008 | 0.029206 | non Marine |
| A | *Diatrypella* | 0.011615 | 0.031143 | non Marine |
| B | *Hygrophorus* | 0.011303 | 0.032541 | non Marine |
| A | *Ascochyta* | 0.011248 | 0.042833 | non Marine |
| A | *Leptodontidium* | 0.011011 | 0.034258 | non Marine |
| A | *Xenocylindrosporium* | 0.010948 | 0.036418 | non Marine |
| A | *Pleurostoma* | 0.010930 | 0.049413 | non Marine |
| A | *Paracladophialophora* | 0.010893 | 0.027247 | non Marine |
| A | *Cladophialophora* | 0.010815 | 0.021026 | non Marine |
| B | *Fomitopsis* | 0.010725 | 0.047170 | non Marine |
| B | *Phellodon* | 0.010711 | 0.038083 | non Marine |
| A | *Lophodermium* | 0.010696 | 0.035478 | non Marine |
| A | *Thyridium* | 0.010637 | 0.030463 | non Marine |
| B | *Sampaiozyma* | 0.010552 | 0.022110 | non Marine |
| A | *Plenodomus* | 0.010535 | 0.035876 | non Marine |
| B | *Basidiodendron* | 0.010418 | 0.052331 | non Marine |
| B | *Resinicium* | 0.010293 | 0.021241 | non Marine |
| A | *Orbilia* | 0.010277 | 0.036698 | non Marine |
| A | *Aequabiliella* | 0.010101 | 0.034700 | non Marine |
| B | *Tilletiopsis* | 0.010059 | 0.041702 | non Marine |
| A | *Arthrocatena* | 0.010000 | 0.028358 | non Marine |
| C | *Chytridiomycota_sp* | 5.278216 | 18.201856 | Not defined |
| F | *Fungi_sp* | 3.707474 | 5.423796 | Not defined |
| A | *Cordycipitaceae_sp* | 0.620622 | 1.513922 | Not defined |
| A | *Didymellaceae_sp* | 0.557818 | 0.879104 | Not defined |
| R | *Rozellomycota_gen_Incertae_sedis* | 0.517309 | 1.370694 | Not defined |
| A | *Pleosporaceae_gen_Incertae_sedis* | 0.240540 | 0.531433 | Not defined |
| B | *Ceratobasidiaceae_sp* | 0.116180 | 0.489720 | Not defined |
| B | *Malasseziales_gen_Incertae_sedis* | 0.088667 | 0.143531 | Not defined |
| A | *Ascomycota_sp* | 0.082441 | 0.126693 | Not defined |
| A | *Sordariomycetes_sp* | 0.078883 | 0.232358 | Not defined |
| B | *Agaricomycetes_sp* | 0.075040 | 0.162971 | Not defined |
| A | *Amplistromataceae_gen_Incertae_sedis* | 0.067503 | 0.147163 | Not defined |
| A | *Myriangiales_gen_Incertae_sedis* | 0.050420 | 0.135120 | Not defined |
| A | *Xylariales_sp* | 0.050202 | 0.142251 | Not defined |
| A | *Leotiomycetes_sp* | 0.046433 | 0.110440 | Not defined |
| A | *Dothideomycetes_sp* | 0.045352 | 0.126368 | Not defined |
| B | *Basidiomycota_sp* | 0.038838 | 0.085151 | Not defined |
| B | *Agaricales_sp* | 0.038321 | 0.069414 | Not defined |
| B | *Cantharellales_sp* | 0.033586 | 0.122578 | Not defined |
| R | *GS11_gen_Incertae_sedis* | 0.031605 | 0.060461 | Not defined |
| A | *Chaetothyriales_sp* | 0.031539 | 0.063304 | Not defined |
| A | *Helotiales_sp* | 0.030044 | 0.093032 | Not defined |
| A | *Pseudeurotiaceae_gen_Incertae_sedis* | 0.029639 | 0.072053 | Not defined |
| B | *Cantharellales_gen_Incertae_sedis* | 0.026075 | 0.076579 | Not defined |
| A | *Hypocreales_sp* | 0.025948 | 0.049480 | Not defined |
| A | *Mycosphaerellaceae_sp* | 0.022636 | 0.086084 | Not defined |
| A | *Phaeosphaeriaceae_gen_Incertae_sedis* | 0.022348 | 0.036220 | Not defined |
| B | *Polyporales_sp* | 0.019452 | 0.088705 | Not defined |
| R | *GS10_gen_Incertae_sedis* | 0.018621 | 0.036145 | Not defined |
| A | *Pyronemataceae_sp* | 0.016708 | 0.058694 | Not defined |
| A | *Phaeomoniellaceae_gen_Incertae_sedis* | 0.016041 | 0.056793 | Not defined |
| B | *Tremellales_gen_Incertae_sedis* | 0.014407 | 0.033919 | Not defined |
| A | *Rhytismataceae_sp* | 0.014001 | 0.071129 | Not defined |
| A | *Clavicipitaceae_sp* | 0.012868 | 0.059710 | Not defined |
| A | *Pleosporales_sp* | 0.012599 | 0.038181 | Not defined |
| A | *Phaeosphaeriaceae_sp* | 0.011847 | 0.046193 | Not defined |
| A | *Diatrypaceae_sp* | 0.011653 | 0.036079 | Not defined |
| A | *Vibrisseaceae_gen_Incertae_sedis* | 0.011609 | 0.068317 | Not defined |
| B | *Trichosporonaceae_sp* | 0.010791 | 0.056348 | Not defined |
| B | *Fomitopsidaceae_gen_Incertae_sedis* | 0.010494 | 0.029191 | Not defined |
| B | *Neofavolus* | 0.009859 | 0.044849 | Rare |
| A | *Verrucocladosporium* | 0.009832 | 0.025439 | Rare |
| B | *Trichaptum* | 0.009824 | 0.043368 | Rare |
| B | *Butlerelfia* | 0.009643 | 0.058656 | Rare |
| B | *Fibricium* | 0.009478 | 0.023591 | Rare |
| B | *Lepiota* | 0.009439 | 0.057415 | Rare |
| B | *Vararia* | 0.009308 | 0.023818 | Rare |
| A | *Apiosordaria* | 0.009290 | 0.056508 | Rare |
| B | *Hannaella* | 0.009274 | 0.023436 | Rare |
| A | *Aspergillaceae_sp* | 0.009257 | 0.025948 | Rare |
| B | *Pterula* | 0.009236 | 0.040132 | Rare |
| A | *Apiosporaceae_gen_Incertae_sedis* | 0.009117 | 0.025546 | Rare |
| A | *Pezizaceae_gen_Incertae_sedis* | 0.009075 | 0.033127 | Rare |
| A | *Clypeosphaeria* | 0.009069 | 0.050057 | Rare |
| B | *Omphalotus* | 0.009015 | 0.049063 | Rare |
| B | *Entoloma* | 0.008970 | 0.047434 | Rare |
| A | *Lophiostoma* | 0.008946 | 0.037745 | Rare |
| A | *Celosporium* | 0.008936 | 0.041577 | Rare |
| A | *Mycoarthris* | 0.008927 | 0.022741 | Rare |
| A | *Arthrinium* | 0.008915 | 0.047869 | Rare |
| B | *Mycoacia* | 0.008903 | 0.027831 | Rare |
| A | *Pyrenochaeta* | 0.008860 | 0.035897 | Rare |
| B | *Postia* | 0.008850 | 0.026959 | Rare |
| A | *Spissiomyces* | 0.008746 | 0.019118 | Rare |
| A | *Alfaria* | 0.008707 | 0.025239 | Rare |
| A | *Saccharomyces* | 0.008691 | 0.032297 | Rare |
| B | *Gloiothele* | 0.008595 | 0.027053 | Rare |
| B | *Fomes* | 0.008541 | 0.019999 | Rare |
| C | *Powellomyces* | 0.008433 | 0.036822 | Rare |
| A | *Cyclaneusma* | 0.008307 | 0.024034 | Rare |
| B | *Entylomataceae_gen_Incertae_sedis* | 0.008306 | 0.024674 | Rare |
| A | *Elsinoe* | 0.008130 | 0.030549 | Rare |
| A | *Dinemasporium* | 0.008048 | 0.022438 | Rare |
| B | *Plicaturopsis* | 0.008006 | 0.034090 | Rare |
| A | *Capnodiales_gen_Incertae_sedis* | 0.007996 | 0.022825 | Rare |
| A | *Melanommataceae_sp* | 0.007995 | 0.032603 | Rare |
| A | *Gnomoniopsis* | 0.007928 | 0.020760 | Rare |
| A | *Herpotrichiellaceae_sp* | 0.007828 | 0.016026 | Rare |
| A | *Hypoxylon* | 0.007781 | 0.016871 | Rare |
| A | *Phomatodes* | 0.007744 | 0.017610 | Rare |
| A | *Lophium* | 0.007676 | 0.036747 | Rare |
| B | *Myxarium* | 0.007657 | 0.022504 | Rare |
| A | *Hymenoscyphus* | 0.007598 | 0.022665 | Rare |
| A | *Beauveria* | 0.007535 | 0.014380 | Rare |
| A | *Hanseniaspora* | 0.007521 | 0.022053 | Rare |
| B | *Polyporus* | 0.007444 | 0.045278 | Rare |
| A | *Pringsheimia* | 0.007421 | 0.019815 | Rare |
| A | *Neodevriesia* | 0.007392 | 0.019684 | Rare |
| B | *Hyphoderma* | 0.007223 | 0.017854 | Rare |
| A | *Sporormiaceae_sp* | 0.007143 | 0.032090 | Rare |
| B | *Flammulina* | 0.007142 | 0.033405 | Rare |
| B | *Pholiota* | 0.007115 | 0.023076 | Rare |
| A | *Melanomma* | 0.007101 | 0.025519 | Rare |
| B | *Hydnodontaceae_sp* | 0.007060 | 0.023084 | Rare |
| A | *Tetracladium* | 0.007020 | 0.019510 | Rare |
| A | *Antarctolichenia* | 0.006922 | 0.021693 | Rare |
| A | *Phacidiopycnis* | 0.006898 | 0.031249 | Rare |
| A | *Microdochium* | 0.006865 | 0.031469 | Rare |
| A | *Arxiella* | 0.006853 | 0.022302 | Rare |
| A | *Sphaeropsis* | 0.006738 | 0.034525 | Rare |
| A | *Ciboria* | 0.006737 | 0.031182 | Rare |
| B | *Coprinopsis* | 0.006723 | 0.023625 | Rare |
| A | *Pectenia* | 0.006662 | 0.040359 | Rare |
| A | *Dipodascaceae_sp* | 0.006651 | 0.021718 | Rare |
| A | *Pleurotheciella* | 0.006631 | 0.040337 | Rare |
| B | *Filobasidiaceae_gen_Incertae_sedis* | 0.006628 | 0.022335 | Rare |
| A | *Neofusicoccum* | 0.006544 | 0.027089 | Rare |
| A | *Septoriella* | 0.006544 | 0.033901 | Rare |
| A | *Protoparmeliopsis* | 0.006528 | 0.024176 | Rare |
| A | *Galactomyces* | 0.006509 | 0.029267 | Rare |
| A | *Glarea* | 0.006502 | 0.021440 | Rare |
| A | *Hendersonia* | 0.006454 | 0.017974 | Rare |
| B | *Heteroacanthella* | 0.006422 | 0.039063 | Rare |
| A | *Uzbekistanica* | 0.006404 | 0.018497 | Rare |
| B | *Moesziomyces* | 0.006257 | 0.023026 | Rare |
| B | *Bulleromyces* | 0.006242 | 0.030148 | Rare |
| B | *Cerrena* | 0.006236 | 0.016649 | Rare |
| B | *Panellus* | 0.006228 | 0.018173 | Rare |
| B | *Subulicystidium* | 0.006222 | 0.019943 | Rare |
| A | *Meristemomyces* | 0.006072 | 0.030858 | Rare |
| B | *Tygervalleyomyces* | 0.006041 | 0.021732 | Rare |
| A | *Hypoderma* | 0.006032 | 0.019794 | Rare |
| A | *Neophaeomoniella* | 0.006006 | 0.026079 | Rare |
| A | *Trichomerium* | 0.005967 | 0.015586 | Rare |
| A | *Pezizella* | 0.005947 | 0.019579 | Rare |
| A | *Amorocoelophoma* | 0.005939 | 0.020201 | Rare |
| A | *Staurothele* | 0.005937 | 0.036114 | Rare |
| A | *Desmazierella* | 0.005883 | 0.033783 | Rare |
| A | *Hyphodiscus* | 0.005881 | 0.017888 | Rare |
| A | *Nothophaeotheca* | 0.005807 | 0.023584 | Rare |
| A | *Sympoventuriaceae_gen_Incertae_sedis* | 0.005786 | 0.019920 | Rare |
| A | *Zygoascus* | 0.005763 | 0.026459 | Rare |
| B | *Papiliotrema* | 0.005673 | 0.016227 | Rare |
| B | *Crepidotus* | 0.005665 | 0.014212 | Rare |
| A | *Physciaceae_sp* | 0.005559 | 0.026410 | Rare |
| A | *Comoclathris* | 0.005550 | 0.013400 | Rare |
| A | *Neopyrenochaeta* | 0.005549 | 0.032737 | Rare |
| A | *Didymosphaeria* | 0.005507 | 0.017865 | Rare |
| A | *Sympoventuriaceae_sp* | 0.005455 | 0.021249 | Rare |
| A | *Biscogniauxia* | 0.005452 | 0.016280 | Rare |
| B | *Tylosporaceae_gen_Incertae_sedis* | 0.005419 | 0.023126 | Rare |
| B | *Oliveonia* | 0.005327 | 0.020967 | Rare |
| B | *Typhula* | 0.005322 | 0.020170 | Rare |
| B | *Ceratobasidiaceae_gen_Incertae_sedis* | 0.005294 | 0.025001 | Rare |
| B | *Phaffia* | 0.005273 | 0.019902 | Rare |
| C | *Halomyces* | 0.005247 | 0.031916 | Rare |
| B | *Phaeophlebiopsis* | 0.005246 | 0.016118 | Rare |
| B | *Junghuhnia* | 0.005170 | 0.029864 | Rare |
| A | *Colletotrichum* | 0.005127 | 0.011562 | Rare |
| A | *Verrucaria* | 0.005114 | 0.024056 | Rare |
| B | *Tomentella* | 0.005109 | 0.013953 | Rare |
| A | *Hormonema* | 0.005097 | 0.025720 | Rare |
| B | *Gloeophyllum* | 0.005050 | 0.022999 | Rare |
| A | *Apenidiella* | 0.005002 | 0.027950 | Rare |
| A | *Sawadaea* | 0.004993 | 0.030368 | Rare |
| A | *Mycosphaerellaceae_gen_Incertae_sedis* | 0.004968 | 0.028402 | Rare |
| B | *Efibula* | 0.004947 | 0.014530 | Rare |
| B | *Colacogloea* | 0.004910 | 0.022735 | Rare |
| A | *Lectera* | 0.004849 | 0.021670 | Rare |
| B | *Pleurotus* | 0.004822 | 0.019054 | Rare |
| A | *Funiliomyces* | 0.004746 | 0.013140 | Rare |
| A | *Ascovirgaria* | 0.004723 | 0.028727 | Rare |
| B | *Sporidiobolus* | 0.004717 | 0.025640 | Rare |
| B | *Meruliopsis* | 0.004684 | 0.014555 | Rare |
| B | *Hymenochaete* | 0.004669 | 0.020068 | Rare |
| A | *Chalastospora* | 0.004618 | 0.020297 | Rare |
| B | *Hapalopilus* | 0.004601 | 0.022787 | Rare |
| B | *Tulostoma* | 0.004567 | 0.014434 | Rare |
| A | *Mycosphaerella* | 0.004508 | 0.015612 | Rare |
| A | *Chaetosphaeronema* | 0.004499 | 0.020842 | Rare |
| A | *Pseudotaeniolina* | 0.004498 | 0.019218 | Rare |
| B | *Trichosporon* | 0.004468 | 0.018449 | Rare |
| B | *Brevicellicium* | 0.004430 | 0.016533 | Rare |
| B | *Abortiporus* | 0.004404 | 0.020786 | Rare |
| A | *Leptosphaeria* | 0.004392 | 0.017787 | Rare |
| A | *Orbiliales_sp* | 0.004387 | 0.023978 | Rare |
| A | *Stachybotryaceae_sp* | 0.004362 | 0.019195 | Rare |
| A | *Myrmecridium* | 0.004326 | 0.018347 | Rare |
| B | *Phaeoclavulina* | 0.004324 | 0.012680 | Rare |
| B | *Lentinellus* | 0.004304 | 0.013424 | Rare |
| A | *Pleosporales_gen_Incertae_sedis* | 0.004300 | 0.026156 | Rare |
| A | *Lachnellula* | 0.004285 | 0.022430 | Rare |
| A | *Rachicladosporium* | 0.004246 | 0.017359 | Rare |
| B | *Pisolithus* | 0.004233 | 0.016459 | Rare |
| B | *Saitozyma* | 0.004204 | 0.014373 | Rare |
| B | *Cyphellopsis* | 0.004128 | 0.025112 | Rare |
| A | *Aspergillaceae_gen_Incertae_sedis* | 0.004124 | 0.012108 | Rare |
| A | *Heterophaeomoniella* | 0.004112 | 0.013550 | Rare |
| A | *Dendryphion* | 0.004083 | 0.021734 | Rare |
| A | *Teratosphaeriaceae_sp* | 0.004049 | 0.019728 | Rare |
| B | *Fibroporia* | 0.004035 | 0.013069 | Rare |
| A | *Ampelomyces* | 0.004022 | 0.023072 | Rare |
| A | *Pseudeurotium* | 0.003928 | 0.012469 | Rare |
| A | *Neobulgaria* | 0.003913 | 0.017348 | Rare |
| A | *Phaeococcomycetaceae_gen_Incertae_sedis* | 0.003892 | 0.019554 | Rare |
| A | *Constantinomyces* | 0.003846 | 0.011769 | Rare |
| B | *Trechisporales_gen_Incertae_sedis* | 0.003796 | 0.011731 | Rare |
| A | *Nigrograna* | 0.003788 | 0.010046 | Rare |
| B | *Golubevia* | 0.003748 | 0.015416 | Rare |
| A | *Tolypocladium* | 0.003737 | 0.014173 | Rare |
| B | *Ceraceomyces* | 0.003706 | 0.013283 | Rare |
| A | *Erysiphaceae_gen_Incertae_sedis* | 0.003670 | 0.022322 | Rare |
| A | *Varicosporellopsis* | 0.003670 | 0.022322 | Rare |
| A | *Setophaeosphaeria* | 0.003656 | 0.015358 | Rare |
| A | *Broomella* | 0.003635 | 0.014472 | Rare |
| A | *Discosia* | 0.003625 | 0.016132 | Rare |
| B | *Moniliella* | 0.003607 | 0.016273 | Rare |
| A | *Paecilomyces* | 0.003600 | 0.018203 | Rare |
| A | *Cistella* | 0.003589 | 0.018024 | Rare |
| C | *Rhizophydiomycetes_gen_Incertae_sedis* | 0.003584 | 0.021144 | Rare |
| A | *Stachybotrys* | 0.003574 | 0.014203 | Rare |
| A | *Moristroma* | 0.003558 | 0.016289 | Rare |
| B | *Calyptella* | 0.003464 | 0.021070 | Rare |
| B | *Gymnopilus* | 0.003464 | 0.013742 | Rare |
| A | *Spegazzinia* | 0.003456 | 0.020176 | Rare |
| B | *Vascellum* | 0.003418 | 0.016737 | Rare |
| A | *Scoliciosporum* | 0.003390 | 0.013362 | Rare |
| B | *Phallus* | 0.003363 | 0.009892 | Rare |
| A | *Collarina* | 0.003358 | 0.011576 | Rare |
| B | *Tomentellopsis* | 0.003356 | 0.020414 | Rare |
| A | *Thyronectria* | 0.003350 | 0.010094 | Rare |
| B | *Udeniomyces* | 0.003294 | 0.020034 | Rare |
| A | *Rhinocladiella* | 0.003283 | 0.007788 | Rare |
| B | *Aurantiporus* | 0.003251 | 0.012080 | Rare |
| A | *Phialophora* | 0.003250 | 0.009471 | Rare |
| A | *Monographella* | 0.003240 | 0.008935 | Rare |
| A | *Gamszarea* | 0.003223 | 0.008676 | Rare |
| A | *Italica* | 0.003211 | 0.019531 | Rare |
| A | *Petrophila* | 0.003198 | 0.013533 | Rare |
| A | *Neocosmospora* | 0.003173 | 0.016529 | Rare |
| R | *Paramicrosporidium* | 0.003172 | 0.009531 | Rare |
| B | *Uncobasidium* | 0.003166 | 0.018464 | Rare |
| A | *Tricladium* | 0.003144 | 0.013826 | Rare |
| A | *Lentithecium* | 0.003143 | 0.016454 | Rare |
| B | *Obba* | 0.003136 | 0.015528 | Rare |
| A | *Trichomeriaceae_sp* | 0.003109 | 0.007564 | Rare |
| A | *Jattaea* | 0.003103 | 0.018878 | Rare |
| B | *Mrakia* | 0.003094 | 0.011125 | Rare |
| R | *Branch03_gen_Incertae_sedis* | 0.003058 | 0.013046 | Rare |
| A | *Parafenestella* | 0.003037 | 0.016920 | Rare |
| B | *Hohenbuehelia* | 0.003028 | 0.012920 | Rare |
| A | *Pleosporaceae_sp* | 0.003013 | 0.013102 | Rare |
| B | *Oxyporus* | 0.002993 | 0.009912 | Rare |
| B | *Cystobasidiomycetes_sp* | 0.002947 | 0.016194 | Rare |
| B | *Cyclocybe* | 0.002946 | 0.017920 | Rare |
| A | *Sporothrix* | 0.002946 | 0.017920 | Rare |
| B | *Kwoniella* | 0.002942 | 0.010438 | Rare |
| B | *Corticium* | 0.002915 | 0.009341 | Rare |
| A | *Densocarpa* | 0.002903 | 0.015931 | Rare |
| A | *Neoroussoella* | 0.002892 | 0.013431 | Rare |
| A | *Bisifusarium* | 0.002874 | 0.012674 | Rare |
| A | *Cylindrodendrum* | 0.002835 | 0.012523 | Rare |
| B | *Sugitazyma* | 0.002832 | 0.017225 | Rare |
| A | *Thelebolaceae_sp* | 0.002827 | 0.013673 | Rare |
| B | *Schizoporaceae_sp* | 0.002784 | 0.007658 | Rare |
| A | *Capitotricha* | 0.002777 | 0.016892 | Rare |
| A | *Glomerobolus* | 0.002752 | 0.016741 | Rare |
| A | *Cladorrhinum* | 0.002733 | 0.008142 | Rare |
| A | *Dendrophoma* | 0.002692 | 0.011587 | Rare |
| B | *Hydnellum* | 0.002678 | 0.010565 | Rare |
| A | *Stromatoseptoria* | 0.002656 | 0.008291 | Rare |
| A | *Teloschistaceae_gen_Incertae_sedis* | 0.002655 | 0.012658 | Rare |
| B | *Heterocephalacria* | 0.002632 | 0.010101 | Rare |
| A | *Dlhawksworthia* | 0.002580 | 0.012533 | Rare |
| A | *Foliophoma* | 0.002561 | 0.010440 | Rare |
| B | *Singerocybe* | 0.002543 | 0.008209 | Rare |
| A | *Sarea* | 0.002523 | 0.009385 | Rare |
| B | *Candolleomyces* | 0.002466 | 0.008499 | Rare |
| A | *Crocicreas* | 0.002455 | 0.007969 | Rare |
| A | *Pseudocyclothyriella* | 0.002453 | 0.011663 | Rare |
| A | *Calycina* | 0.002453 | 0.007059 | Rare |
| B | *Hebeloma* | 0.002451 | 0.008646 | Rare |
| A | *Vermiconia* | 0.002445 | 0.008440 | Rare |
| A | *Fuscostagonospora* | 0.002431 | 0.008998 | Rare |
| B | *Fibrodontia* | 0.002397 | 0.012190 | Rare |
| B | *Irpex* | 0.002394 | 0.012660 | Rare |
| A | *Ameghiniella* | 0.002376 | 0.008362 | Rare |
| A | *Teratosphaeriaceae_gen_Incertae_sedis* | 0.002373 | 0.014433 | Rare |
| B | *Limacella* | 0.002358 | 0.014345 | Rare |
| B | *Leucosporidium* | 0.002336 | 0.010974 | Rare |
| A | *Gliocladiopsis* | 0.002331 | 0.013390 | Rare |
| A | *Ilyonectria* | 0.002311 | 0.014055 | Rare |
| A | *Acidomyces* | 0.002303 | 0.014006 | Rare |
| A | *Arthoniomycetes_gen_Incertae_sedis* | 0.002300 | 0.008811 | Rare |
| A | *Ceratostomella* | 0.002295 | 0.012194 | Rare |
| A | *Dissoconium* | 0.002280 | 0.007172 | Rare |
| A | *Fusicladium* | 0.002263 | 0.008241 | Rare |
| B | *Pseudobaeospora* | 0.002254 | 0.009917 | Rare |
| B | *Phlebiopsis* | 0.002251 | 0.008815 | Rare |
| B | *Inocybe* | 0.002235 | 0.013594 | Rare |
| A | *Wickerhamiella* | 0.002235 | 0.013594 | Rare |
| A | *Boeremia* | 0.002221 | 0.007894 | Rare |
| A | *Niesslia* | 0.002211 | 0.013449 | Rare |
| B | *Dichomitus* | 0.002207 | 0.006422 | Rare |
| B | *Bovista* | 0.002207 | 0.008812 | Rare |
| A | *Neostagonospora* | 0.002193 | 0.009314 | Rare |
| B | *Kavinia* | 0.002171 | 0.007759 | Rare |
| A | *Xylariales_gen_Incertae_sedis* | 0.002165 | 0.011285 | Rare |
| A | *Dothidea* | 0.002150 | 0.009810 | Rare |
| B | *Confertobasidium* | 0.002149 | 0.010317 | Rare |
| B | *Phloeomana* | 0.002138 | 0.006627 | Rare |
| A | *Letendraea* | 0.002137 | 0.006517 | Rare |
| B | *Athelia* | 0.002131 | 0.009290 | Rare |
| A | *Coniella* | 0.002129 | 0.011961 | Rare |
| A | *Nonappendiculata* | 0.002117 | 0.009543 | Rare |
| A | *Dothiorella* | 0.002092 | 0.012723 | Rare |
| A | *Protofenestella* | 0.002090 | 0.007982 | Rare |
| A | *Mycosphaerellales_sp* | 0.002086 | 0.007147 | Rare |
| B | *Lactarius* | 0.002066 | 0.012564 | Rare |
| B | *Neoantrodia* | 0.002064 | 0.012552 | Rare |
| A | *Lasionectriopsis* | 0.002060 | 0.007350 | Rare |
| B | *Fomitopsidaceae_sp* | 0.002054 | 0.010141 | Rare |
| B | *Atheliales_sp* | 0.002040 | 0.010252 | Rare |
| A | *Valsaria* | 0.001985 | 0.007659 | Rare |
| A | *Loramycetaceae_sp* | 0.001964 | 0.006194 | Rare |
| B | *Paralepista* | 0.001957 | 0.006693 | Rare |
| A | *Pseudoarthrographis* | 0.001951 | 0.008809 | Rare |
| A | *Hyaloscyphaceae_sp* | 0.001947 | 0.009703 | Rare |
| B | *Craterellus* | 0.001938 | 0.010780 | Rare |
| B | *Trechisporales_sp* | 0.001896 | 0.011534 | Rare |
| A | *Chordomyces* | 0.001891 | 0.009642 | Rare |
| B | *Sporidiobolales_gen_Incertae_sedis* | 0.001881 | 0.005570 | Rare |
| A | *Harmoniella* | 0.001864 | 0.010163 | Rare |
| A | *Hypomyces* | 0.001824 | 0.010115 | Rare |
| B | *Coprinus* | 0.001809 | 0.008365 | Rare |
| B | *Rhodocybe* | 0.001790 | 0.006262 | Rare |
| A | *Pleurophoma* | 0.001783 | 0.008264 | Rare |
| A | *Pseudoanungitea* | 0.001762 | 0.008274 | Rare |
| B | *Grifola* | 0.001761 | 0.010711 | Rare |
| B | *Litschauerella* | 0.001756 | 0.008748 | Rare |
| B | *Typhulaceae_sp* | 0.001745 | 0.009130 | Rare |
| B | *Amanita* | 0.001740 | 0.009066 | Rare |
| B | *Geastrum* | 0.001738 | 0.006924 | Rare |
| A | *Candelariella* | 0.001735 | 0.010555 | Rare |
| B | *Lyophyllum* | 0.001718 | 0.008626 | Rare |
| A | *Idriella* | 0.001700 | 0.007396 | Rare |
| A | *Acrocalymma* | 0.001694 | 0.010304 | Rare |
| A | *Vermiculariopsiella* | 0.001693 | 0.010299 | Rare |
| A | *Pseudocoleophoma* | 0.001682 | 0.008751 | Rare |
| B | *Skvortzovia* | 0.001648 | 0.008548 | Rare |
| B | *Auriculariales_sp* | 0.001643 | 0.007918 | Rare |
| A | *Halosarpheia* | 0.001635 | 0.007117 | Rare |
| A | *Yamadazyma* | 0.001627 | 0.009898 | Rare |
| A | *Leptosillia* | 0.001600 | 0.004950 | Rare |
| A | *Zeloasperisporium* | 0.001580 | 0.008143 | Rare |
| B | *Chrysozymaceae_gen_Incertae_sedis* | 0.001573 | 0.009569 | Rare |
| B | *Meruliaceae_gen_Incertae_sedis* | 0.001568 | 0.007397 | Rare |
| B | *Macrotyphula* | 0.001567 | 0.005357 | Rare |
| B | *Geminibasidium* | 0.001559 | 0.009484 | Rare |
| A | *Geopyxis* | 0.001558 | 0.009475 | Rare |
| B | *Physisporinus* | 0.001548 | 0.009414 | Rare |
| A | *Mollisina* | 0.001535 | 0.006929 | Rare |
| A | *Nemania* | 0.001530 | 0.005392 | Rare |
| A | *Xylaria* | 0.001530 | 0.008532 | Rare |
| A | *Dictyosporiaceae_gen_Incertae_sedis* | 0.001525 | 0.006191 | Rare |
| A | *Beltrania* | 0.001510 | 0.007151 | Rare |
| A | *Fraxinicola* | 0.001508 | 0.006897 | Rare |
| B | *Donkioporia* | 0.001507 | 0.006421 | Rare |
| A | *Bimuria* | 0.001492 | 0.009073 | Rare |
| A | *Elaphomyces* | 0.001480 | 0.006326 | Rare |
| A | *Montagnula* | 0.001474 | 0.008966 | Rare |
| B | *Auriscalpium* | 0.001468 | 0.008931 | Rare |
| A | *Asterinales_sp* | 0.001461 | 0.008890 | Rare |
| A | *Amandinea* | 0.001460 | 0.005771 | Rare |
| B | *Infundibulicybe* | 0.001441 | 0.008045 | Rare |
| A | *Preussia* | 0.001439 | 0.005550 | Rare |
| A | *Alatospora* | 0.001429 | 0.008694 | Rare |
| M | *Mortierella* | 0.001426 | 0.006525 | Rare |
| A | *Limtongozyma* | 0.001422 | 0.008651 | Rare |
| A | *Bionectriaceae_gen_Incertae_sedis* | 0.001407 | 0.006184 | Rare |
| B | *Clitocybula* | 0.001400 | 0.008518 | Rare |
| A | *Brachyphoris* | 0.001395 | 0.008486 | Rare |
| A | *Toxicocladosporium* | 0.001382 | 0.005866 | Rare |
| A | *Kluyveromyces* | 0.001357 | 0.007080 | Rare |
| A | *Didymosphaeriaceae_sp* | 0.001354 | 0.008239 | Rare |
| A | *Hirsutella* | 0.001353 | 0.005891 | Rare |
| A | *Conioscypha* | 0.001341 | 0.005974 | Rare |
| A | *Sordariales_sp* | 0.001329 | 0.008082 | Rare |
| A | *Anthracina* | 0.001328 | 0.004324 | Rare |
| A | *Fenestella* | 0.001315 | 0.005678 | Rare |
| B | *Solicoccozyma* | 0.001299 | 0.007903 | Rare |
| B | *Dermoloma* | 0.001287 | 0.007827 | Rare |
| B | *Fomitiporia* | 0.001279 | 0.006498 | Rare |
| A | *Chloroscypha* | 0.001275 | 0.007755 | Rare |
| B | *Cartilosoma* | 0.001265 | 0.005453 | Rare |
| A | *Capturomyces* | 0.001255 | 0.005566 | Rare |
| A | *Microscypha* | 0.001247 | 0.004458 | Rare |
| A | *Gibellulopsis* | 0.001220 | 0.007423 | Rare |
| A | *Polyscytalum* | 0.001219 | 0.007415 | Rare |
| B | *Tubaria* | 0.001197 | 0.005856 | Rare |
| A | *Sedecimiella* | 0.001196 | 0.007273 | Rare |
| A | *Oidiodendron* | 0.001195 | 0.006316 | Rare |
| A | *Nectriaceae_sp* | 0.001194 | 0.003894 | Rare |
| A | *Gonatophragmium* | 0.001185 | 0.007209 | Rare |
| A | *Ramimonilia* | 0.001185 | 0.007209 | Rare |
| A | *Fusariella* | 0.001178 | 0.003807 | Rare |
| A | *Pyrenopeziza* | 0.001176 | 0.004992 | Rare |
| B | *Stereaceae_sp* | 0.001162 | 0.006304 | Rare |
| A | *Drepanopeziza* | 0.001143 | 0.005503 | Rare |
| A | *Volutella* | 0.001143 | 0.005284 | Rare |
| B | *Holocotylon* | 0.001117 | 0.006797 | Rare |
| B | *Picipes* | 0.001117 | 0.006797 | Rare |
| A | *Pseudocamaropycnis* | 0.001117 | 0.006797 | Rare |
| B | *Microbotryales_gen_Incertae_sedis* | 0.001062 | 0.006462 | Rare |
| A | *Nodulisporium* | 0.001062 | 0.006462 | Rare |
| A | *Diddensiella* | 0.001053 | 0.004192 | Rare |
| A | *Chaetosphaeria* | 0.001045 | 0.006357 | Rare |
| B | *Rubroboletus* | 0.001032 | 0.006276 | Rare |
| B | *Melampsora* | 0.001032 | 0.006276 | Rare |
| A | *Vexillomyces* | 0.001020 | 0.004667 | Rare |
| A | *Dothiora* | 0.001016 | 0.006179 | Rare |
| B | *Cinereomyces* | 0.001005 | 0.003558 | Rare |
| A | *Xenoanthostomella* | 0.000992 | 0.004445 | Rare |
| A | *Taeniolella* | 0.000984 | 0.004590 | Rare |
| M | *Mortierellaceae_gen_Incertae_sedis* | 0.000982 | 0.005973 | Rare |
| A | *Extremus* | 0.000976 | 0.005188 | Rare |
| A | *Venturia* | 0.000968 | 0.005890 | Rare |
| A | *Metarhizium* | 0.000961 | 0.004283 | Rare |
| A | *Pilidium* | 0.000951 | 0.004996 | Rare |
| A | *Chaetopsina* | 0.000948 | 0.005767 | Rare |
| B | *Ramaria* | 0.000948 | 0.005767 | Rare |
| A | *Atrocalyx* | 0.000934 | 0.003963 | Rare |
| A | *Hypocreales_gen_Incertae_sedis* | 0.000930 | 0.005657 | Rare |
| A | *Stylonectria* | 0.000921 | 0.005601 | Rare |
| A | *Neurospora* | 0.000916 | 0.003849 | Rare |
| B | *Agaricus* | 0.000914 | 0.005561 | Rare |
| B | *Helicogloea* | 0.000911 | 0.003526 | Rare |
| A | *Schizothecium* | 0.000891 | 0.005421 | Rare |
| A | *Xenopolyscytalum* | 0.000880 | 0.005355 | Rare |
| A | *Magnohelicospora* | 0.000880 | 0.005355 | Rare |
| A | *Cryptosphaeria* | 0.000873 | 0.004118 | Rare |
| B | *Myochromella* | 0.000856 | 0.002940 | Rare |
| A | *Gibellula* | 0.000847 | 0.005149 | Rare |
| B | *Sebacinales_sp* | 0.000847 | 0.005149 | Rare |
| B | *Entyloma* | 0.000845 | 0.003741 | Rare |
| B | *Serpula* | 0.000830 | 0.005051 | Rare |
| A | *Crassiclypeus* | 0.000822 | 0.003580 | Rare |
| B | *Lycoperdaceae_sp* | 0.000813 | 0.004943 | Rare |
| B | *Odonticium* | 0.000813 | 0.004943 | Rare |
| B | *Kurtzmanomyces* | 0.000792 | 0.002749 | Rare |
| R | *GS08_gen_Incertae_sedis* | 0.000768 | 0.002698 | Rare |
| B | *Microsporomycetaceae_gen_Incertae_sedis* | 0.000745 | 0.004531 | Rare |
| A | *Pseudocercosporella* | 0.000744 | 0.004524 | Rare |
| A | *Peltigera* | 0.000744 | 0.004524 | Rare |
| A | *Sclerotiniaceae_sp* | 0.000744 | 0.004524 | Rare |
| A | *Biappendiculispora* | 0.000740 | 0.003207 | Rare |
| B | *Rhizoctonia* | 0.000730 | 0.003552 | Rare |
| A | *Lulworthia* | 0.000720 | 0.003063 | Rare |
| A | *Phyllosticta* | 0.000711 | 0.004325 | Rare |
| A | *Sphaerostilbella* | 0.000707 | 0.004303 | Rare |
| A | *Lophiostomataceae_sp* | 0.000706 | 0.003568 | Rare |
| B | *Deconica* | 0.000703 | 0.003920 | Rare |
| A | *Phyllachora* | 0.000698 | 0.004243 | Rare |
| A | *Phragmocephala* | 0.000686 | 0.004170 | Rare |
| A | *Lachnum* | 0.000678 | 0.003407 | Rare |
| A | *Saccothecium* | 0.000677 | 0.004119 | Rare |
| A | *Endoconidioma* | 0.000677 | 0.004119 | Rare |
| A | *Trapeliopsis* | 0.000677 | 0.004119 | Rare |
| A | *Sphaerellopsis* | 0.000661 | 0.002850 | Rare |
| A | *Rhytisma* | 0.000643 | 0.003913 | Rare |
| A | *Kazachstania* | 0.000643 | 0.003913 | Rare |
| A | *Clarireedia* | 0.000631 | 0.003839 | Rare |
| A | *Nothophaeomoniella* | 0.000610 | 0.003708 | Rare |
| A | *Lecanora* | 0.000610 | 0.003708 | Rare |
| A | *Pragmopora* | 0.000610 | 0.003708 | Rare |
| A | *Starmerella* | 0.000605 | 0.002833 | Rare |
| B | *Libkindia* | 0.000604 | 0.002969 | Rare |
| A | *Helotiaceae_sp* | 0.000603 | 0.002276 | Rare |
| A | *Myrmecridiales_gen_Incertae_sedis* | 0.000602 | 0.003662 | Rare |
| A | *Albotricha* | 0.000598 | 0.003637 | Rare |
| B | *Thanatephorus* | 0.000596 | 0.002542 | Rare |
| B | *Leucogyrophana* | 0.000589 | 0.002632 | Rare |
| A | *Roesleria* | 0.000576 | 0.003502 | Rare |
| A | *Phialea* | 0.000576 | 0.003502 | Rare |
| A | *Bacidina* | 0.000567 | 0.003447 | Rare |
| A | *Tympanis* | 0.000567 | 0.003447 | Rare |
| A | *Hypomontagnella* | 0.000565 | 0.003435 | Rare |
| A | *Cyberlindnera* | 0.000558 | 0.002812 | Rare |
| A | *Setoseptoria* | 0.000548 | 0.003336 | Rare |
| B | *Ramariopsis* | 0.000542 | 0.003296 | Rare |
| B | *Teunia* | 0.000542 | 0.003296 | Rare |
| A | *Acarospora* | 0.000542 | 0.003296 | Rare |
| A | *Brachysporium* | 0.000542 | 0.003296 | Rare |
| A | *Ascocoryne* | 0.000541 | 0.002603 | Rare |
| A | *Parathyridaria* | 0.000540 | 0.002729 | Rare |
| B | *Clavulinaceae_gen_Incertae_sedis* | 0.000531 | 0.003233 | Rare |
| A | *Lecanorales_gen_Incertae_sedis* | 0.000531 | 0.003233 | Rare |
| B | *Trichosporonaceae_gen_Incertae_sedis* | 0.000531 | 0.003231 | Rare |
| A | *Nectria* | 0.000531 | 0.003231 | Rare |
| A | *Ophiostoma* | 0.000529 | 0.001994 | Rare |
| A | *Gnomonia* | 0.000518 | 0.002294 | Rare |
| B | *Microbotryozyma* | 0.000511 | 0.002335 | Rare |
| B | *Meira* | 0.000508 | 0.003090 | Rare |
| A | *Phaeoannellomyces* | 0.000508 | 0.003090 | Rare |
| A | *Pseudocosmospora* | 0.000508 | 0.003090 | Rare |
| A | *Pseudocercospora* | 0.000507 | 0.002405 | Rare |
| A | *Kuraishia* | 0.000505 | 0.002391 | Rare |
| B | *Erythrobasidiales_gen_Incertae_sedis* | 0.000502 | 0.001968 | Rare |
| B | *Sebacina* | 0.000498 | 0.003031 | Rare |
| A | *Trichocomaceae_gen_Incertae_sedis* | 0.000498 | 0.003031 | Rare |
| A | *Xenosonderhenia* | 0.000496 | 0.003016 | Rare |
| B | *Entolomataceae_gen_Incertae_sedis* | 0.000495 | 0.002338 | Rare |
| A | *Coniothyrium* | 0.000474 | 0.002884 | Rare |
| A | *Halobyssothecium* | 0.000474 | 0.002884 | Rare |
| A | *Sorocybe* | 0.000474 | 0.002884 | Rare |
| A | *Catillaria* | 0.000474 | 0.002884 | Rare |
| A | *Lasionectria* | 0.000474 | 0.002884 | Rare |
| A | *Thelebolales_gen_Incertae_sedis* | 0.000465 | 0.002829 | Rare |
| A | *Pleurothecium* | 0.000460 | 0.002800 | Rare |
| A | *Tintelnotia* | 0.000440 | 0.002678 | Rare |
| A | *Torulaspora* | 0.000440 | 0.002678 | Rare |
| A | *Breviappendix* | 0.000440 | 0.002678 | Rare |
| A | *Rosellinia* | 0.000440 | 0.002678 | Rare |
| A | *Xylariaceae_sp* | 0.000440 | 0.002678 | Rare |
| A | *Sagenomella* | 0.000427 | 0.001486 | Rare |
| A | *Metapochonia* | 0.000425 | 0.002585 | Rare |
| A | *Microascales_gen_Incertae_sedis* | 0.000425 | 0.002585 | Rare |
| A | *Biatriosporaceae_sp* | 0.000406 | 0.002472 | Rare |
| A | *Morinia* | 0.000406 | 0.002472 | Rare |
| A | *Setophoma* | 0.000399 | 0.002424 | Rare |
| A | *Valsonectria* | 0.000399 | 0.002424 | Rare |
| B | *Phaeotremella* | 0.000390 | 0.002370 | Rare |
| A | *Archaeorhizomyces* | 0.000390 | 0.002370 | Rare |
| B | *Suillus* | 0.000372 | 0.002266 | Rare |
| B | *Meripilus* | 0.000372 | 0.002266 | Rare |
| B | *Farysia* | 0.000372 | 0.002266 | Rare |
| A | *Marquandomyces* | 0.000372 | 0.002266 | Rare |
| A | *Circinotrichum* | 0.000372 | 0.002266 | Rare |
| A | *Magnibotryascoma* | 0.000368 | 0.001627 | Rare |
| A | *Cyphellophoraceae_gen_Incertae_sedis* | 0.000365 | 0.002222 | Rare |
| A | *Globoramichloridium* | 0.000354 | 0.002154 | Rare |
| A | *Parmelina* | 0.000354 | 0.002154 | Rare |
| A | *GS35_ord_Incertae_sedis_gen_Incertae_sedis* | 0.000343 | 0.002085 | Rare |
| A | *Polyphilus* | 0.000343 | 0.002085 | Rare |
| A | *Dimorphospora* | 0.000343 | 0.002085 | Rare |
| A | *Thelonectria* | 0.000343 | 0.002085 | Rare |
| B | *Russula* | 0.000339 | 0.002060 | Rare |
| A | *Acericola* | 0.000335 | 0.001617 | Rare |
| B | *Waitea* | 0.000332 | 0.002020 | Rare |
| A | *Phaeomoniella* | 0.000308 | 0.001322 | Rare |
| A | *Keissleriella* | 0.000262 | 0.001136 | Rare |
| A | *Chaetosphaeriaceae_gen_Incertae_sedis* | 0.000236 | 0.001438 | Rare |
| A | *Microascus* | 0.000230 | 0.001116 | Rare |
| A | *Graphium* | 0.000223 | 0.000947 | Rare |
| A | *Boliniales_gen_Incertae_sedis* | 0.000217 | 0.001319 | Rare |

**Supplementary Table S2** Fungal genera detected either with both 18S V4 and ITS1 barcodes, or with only one of the two in C1 surface samples

| **Barcode** | **Number** | **Genera** |
| --- | --- | --- |
| 18S V4 and ITS1 | 31 | *Gymnopus, Ustilago, Sakaguchia, Sistotrema, Vishniacozyma, Dioszegia, Schizophyllum, Rhodotorula, Auricularia, Kluyveromyces, Ochroconis, Metschnikowia, Suillus, Sugitazyma, Kondoa, Trechispora, Saccharomyces, Cystobasidium, Phlebia, Paramicrosporidium, Hyphodontia, Colacogloea, Kurtzmanomyces, Junghuhnia, Malassezia, Symmetrospora, Exophiala, Torulaspora, Debaryomyces, Gnomonia, Apiotrichum* |
| ITS1 only | 582 | *Clitocybe, Botryobasidium, Acremonium, Lachnellula, Trichosporon, Meristemomyces, Cinereomyces, Trichaptum, Teunia, Geminibasidium, Hypomontagnella, Erysiphe, Cyberlindnera, Periconia, Hapalopilus, Pseudocamaropycnis, Meira, Arthrinium, Varicosporellopsis, Biappendiculispora, Amanita, Mycoarthris, Flammulina, Stromatoseptoria, Angustimassarina, Fraxinicola, Neurospora, Cryptomarasmius, Rhytisma, Nigrospora, Postia, Septoria, Omphalotus, Agaricus, Oidiodendron, Leptosphaeria, Cistella, Pisolithus, Arthrocatena, Kazachstania, Odonticium, Hydnellum, Sarea, Tubulicrinis, Pleurophoma, Nothophaeotheca, Chalastospora, Salinomyces, Idriella, Phragmocephala, Ceratobasidium, Funiliomyces, Confertobasidium, Buckleyzyma, Phaeoclavulina, Sphaeropsis, Ramaria, Pseudotaeniolina, Leucogyrophana, Exidia, Resupinatus, Hypoxylon, Myrothecium, Coprinopsis, Craterellus, Anthracina, Thanatephorus, Gloeophyllum, Acarospora, Uzbekistanica, Dothiora, Gloeoporus, Lophiostoma, Aurantiporus, Bovista, Neofusicoccum, Lophium, Letendraea, Drepanopeziza, Torula, Mycena, Chaetosphaeronema, Microscypha, Itersonilia, Phialophora, Lyophyllum, Talaromyces, Phyllachora, Efibula, Naganishia, Ascochyta, Valsonectria, Betamyces, Endosporium, Glomerobolus, Meruliopsis, Lenzites, Fusarium, Phaffia, Biscogniauxia, Pringsheimia, Nonappendiculata, Pseudocyclothyriella, Pleurostoma, Xenasmatella, Amyloporia, Heteroacanthella, Xanthocarpia, Steccherinum, Brachysporium, Glarea, Phaeophlebiopsis, Elsinoe, Nothophaeomoniella, Constantinomyces, Phialea, Fusicolla, Mortierella, Naevala, Aureobasidium, Sporobolomyces, Pseudoanungitea, Hyphoderma, Bisifusarium, Nigrograna, Phanerochaete, Diddensiella, Myrmecridium, Lachnum, Ophiognomonia, Kwoniella, Moniliella, Alatospora, Valsaria, Dermoloma, Graphium, Phialocephala, Hymenochaete, Abortiporus, Gnomoniopsis, Thyronectria, Psathyrella, Paralepista, Flavoplaca, Limtongozyma, Lepista, Eutypella, Articulospora, Coprinellus, Neovaginatispora, Polyphilus, Hygrophorus, Pseudeurotium, Clypeosphaeria, Hannaella, Acidomyces, Hortaea, Verrucaria, Morinia, Golubevia, Rhizoctonia, Chaetopsina, Calycina, Ceriporia, Kalmusia, Picipes, Hirsutella, Donkioporia, Rachicladosporium, Halobyssothecium, Setoseptoria, Hymenoscyphus, Paraphoma, Erythrobasidium, Phacidiopycnis, Lycoperdon, Tricladium, Sphaerulina, Ascocoryne, Fusicladium, Zygoascus, Zeloasperisporium, Skeletocutis, Cylindrodendrum, Phallus, Circinotrichum, Clydaea, Leptosillia, Xenocylindrosporium, Thelonectria, Ascovirgaria, Dendrophoma, Dlhawksworthia, Starmerella, Cadophora, Oliveonia, Armillaria, Roesleria, Trapeliopsis, Neoroussoella, Dinemasporium, Sistotremastrum, Gibellula, Xenodidymella, Paraphaeosphaeria, Zalaria, Melanomma, Rhodocybe, Monographella, Coniosporium, Ceraceomyces, Peniophora, Radulomyces, Oxyporus, Deconica, Fibroporia, Halosarpheia, Phaeoannellomyces, Sclerostagonospora, Ameghiniella, Geastrum, Pezizella, Magnohelicospora, Peroneutypa, Cutaneotrichosporon, Cyphellophora, Vexillomyces, Lentinellus, Dimorphospora, Ophiostoma, Rosellinia, Resinicium, Neopyrenochaeta, Neosetophoma, Comoclathris, Dendryphion, Preussia, Neocucurbitaria, Myxarium, Myochromella, Fomitopsis, Capturomyces, Macrotyphula, Farysia, Lasionectriopsis, Uncobasidium, Elaphomyces, Ganoderma, Setophoma, Neophaeococcomyces, Sphaerellopsis, Vascellum, Pseudocosmospora, Mycoacia, Verrucocladosporium, Phlebiopsis, Hanseniaspora, Hypomyces, Dissoconium, Nemania, Fuscostagonospora, Ciboria, Protoparmeliopsis, Aequabiliella, Botrytis, Litschauerella, Waitea, Acericola, Gibellulopsis, Celosporium, Brachyphoris, Neocosmospora, Microdochium, Skvortzovia, Discosia, Neobulgaria, Septoriella, Chaetomium, Scolecobasidium, Setophaeosphaeria, Cortinarius, Subulicystidium, Pseudorobillarda, Gibberella, Lentithecium, Sampaiozyma, Cyphellopsis, Broomella, Leucosporidium, Fusariella, Galactomyces, Pseudopithomyces, Cabalodontia, Bullera, Phaeomoniella, Heterotruncatella, Taeniolella, Pseudoarthrographis, Exidiopsis, Sedecimiella, Piskurozyma, Blumeria, Gamszarea, Genolevuria, Fomitiporia, Phloeomana, Xylodon, Iodophanus, Peniophorella, Serpula, Cyclaneusma, Xylaria, Cladosporium, Mollisina, Diatrypella, Eutypa, Sorocybe, Pterula, Gloiothele, Stereum, Sidera, Gliocladiopsis, Neocatenulostroma, Bartalinia, Curvularia, Plicaturopsis, Alfaria, Ampelomyces, Ceratostomella, Cryptosphaeria, Gonatophragmium, Diatrype, Phellodon, Fonsecazyma, Teichospora, Mrakia, Trichomerium, Hendersonia, Taphrina, Paracladophialophora, Dichomitus, Parastagonospora, Xenoanthostomella, Hydnum, Ochrocladosporium, Marquandomyces, Phaeoacremonium, Rhinocladiella, Tintelnotia, Lactarius, Magnibotryascoma, Vararia, Pyrenochaeta, Fenestella, Lophodermium, Neoascochyta, Cerrena, Mollisia, Pseudobaeospora, Rhodosporidiobolus, Bjerkandera, Hohenbuehelia, Boeremia, Acrodontium, Candida, Italica, Candolleomyces, Paraophiobolus, Saitozyma, Pyrenochaetopsis, Emericellopsis, Russula, Breviappendix, Gymnopilus, Cladorrhinum, Hormonema, Panellus, Tricellula, Sporothrix, Cylindrobasidium, Phaeosphaeria, Papiliotrema, Vermiculariopsiella, Scopuloides, Trametes, Ramimonilia, Byssomerulius, Lecanora, Moesziomyces, Phaeoisaria, Bulleromyces, Thyridium, Phaeosphaeriopsis, Catillaria, Neophaeomoniella, Kuraishia, Yamadazyma, Alternaria, Holocotylon, Cryptococcus, Amorocoelophoma, Paraconiothyrium, Phomatospora, Keissleriella, Chordomyces, Paecilomyces, Wickerhamiella, Tulostoma, Coniochaeta, Chaetosphaeria, Meripilus, Didymocyrtis, Dothidea, Tolypocladium, Sertulicium, Basidiodendron, Beltrania, Porostereum, Neodevriesia, Volutella, Heterobasidion, Metarhizium, Typhula, Knufia, Pectenia, Microbotryozyma, Collarina, Cytospora, Hypoderma, Libkindia, Phomatodes, Atrocalyx, Stagonosporopsis, Pseudocoleophoma, Purpureocillium, Effuseotrichosporon, Epicoccum, Tygervalleyomyces, Fuscoporia, Leptospora, Arxiella, Pseudocercospora, Brevicellicium, Petrophila, Gloeocystidiellum, Foliophoma, Lectera, Tubaria, Polyscytalum, Corticium, Xenopolyscytalum, Acrocalymma, Plenodomus, Cylindrium, Filobasidium, Entoloma, Densocarpa, Microascus, Irpex, Parafenestella, Ramariopsis, Powellomyces, Tomentellopsis, Coniothyrium, Pholiota, Tetracladium, Cladophialophora, Antarctolichenia, Trichoderma, Leptodontidium, Stemphylium, Staurothele, Colletotrichum, Amphinema, Hebeloma, Rhodocollybia, Plectosphaerella, Mycosphaerella, Cryptostroma, Pseudozyma, Burgoa, Pseudoophiobolus, Scoliciosporum, Tomentella, Montagnula, Orbilia, Halomyces, Ceriporiopsis, Lasionectria, Sawadaea, Cyanosporus, Penicillium, Obba, Cartilosoma, Grifola, Cyclocybe, Fomes, Incrucipulum, Harmoniella, Spissiomyces, Vermiconia, Sarocladium, Geopyxis, Saccothecium, Lecanicillium, Coriolopsis, Sagenomella, Aspergillus, Clarireedia, Endoconidioma, Crepidotus, Pyrenopeziza, Pleurotus, Coprinus, Phyllosticta, Heterocephalacria, Inocybe, Sterigmatomyces, Beauveria, Erratomyces, Amyloxenasma, Hyphodiscus, Cryptovalsa, Athelia, Apiosordaria, Auriscalpium, Schizothecium, Spegazzinia, Neofavolus, Parengyodontium, Vuilleminia, Paragibellulopsis, Pilidium, Neostagonospora, Coniella, Lapidomyces, Curvibasidium, Fibricium, Protofenestella, Crocicreas, Pragmopora, Tilletiopsis, Amandinea, Hypholoma, Plectania, Solicoccozyma, Albotricha, Entyloma, Kavinia, Jattaea, Phaeococcomyces, Podosphaera, Wallemia, Hyphodermella, Bimuria, Sporidiobolus, Neophaeotheca, Conioscypha, Heterophaeomoniella, Rigidoporus, Fibrodontia, Ramularia, Lepiota, Strobilurus, Desmazierella, Singerocybe, Baeospora, Amylostereum, Extremus, Apenidiella, Crassiclypeus, Daedaleopsis, Moristroma, Thelebolus, Cystofilobasidium, Didymosphaeria, Sebacina, Helicogloea, Perusta, Tausonia, Tricholoma, Didymella* |
| 18S V4 only | 9 | *Rozella, Olpidium, Quambalaria, Geotrichum, Issatchenkia, Nowakowskiella, Oberwinklerozyma, Gjaerumia, Rhizophydium* |
